# The multifaceted roles of the transcriptional coactivator TAZ in extravillous trophoblast development of the human placenta

**DOI:** 10.1073/pnas.2426385122

**Published:** 2025-04-14

**Authors:** Gudrun Meinhardt, Hanna Waldhäusl, Andreas I. Lackner, Jasmin Wächter, Theresa Maxian, Anna-Lena Höbler, Sigrid Vondra, Victoria Kunihs, Leila Saleh, Peter Haslinger, Peter Kiraly, Andras Szilagyi, Nandor G. Than, Jürgen Pollheimer, Sandra Haider, Martin Knöfler

**Affiliations:** ^a^Placental Development Group, Reproductive Biology Unit, Department of Obstetrics and Gynaecology, Medical University of Vienna, Vienna A-1090, Austria; ^b^Maternal-Fetal Immunology Group, Reproductive Biology Unit, Department of Obstetrics and Gynaecology, Medical University of Vienna, Vienna A-1090, Austria; ^c^Systems Biology of Reproduction Lendulet Group, Institute of Molecular Life Sciences, Hungarian Research Network (HUN-REN) Research Centre for Natural Sciences, Budapest 1117, Hungary; ^d^Maternity Private Clinic of Obstetrics and Gynecology, Budapest 1126, Hungary; ^e^Department of Obstetrics and Gynecology, Semmelweis University, Budapest 1088, Hungary

**Keywords:** human placenta, extravillous trophoblast, trophoblast organoid, TAZ, differentiation

## Abstract

Insights into the molecular processes that drive early development of the human placenta is crucial for our understanding of pregnancy complications such as preeclampsia and fetal growth restriction, since defects in maturation of its epithelial cell, the trophoblast, have been detected in the severe forms of these diseases. However, key regulators specifying the differentiated trophoblast subtypes of the placenta are only slowly emerging. By using diverse trophoblast cell models, we herein show that the transcriptional coactivator of HIPPO signaling, TAZ, plays a pivotal role in the development of invasive extravillous trophoblasts (EVTs), cells that are essential for decidual vessel remodeling and adaption of maternal blood flow to the placenta. Ribonucleic acid sequencing (RNA-seq) or protein analyses upon TAZ gene silencing or CRISPR-Cas9-mediated knockout in differentiating trophoblast stem cells, organoids, primary EVTs, choriocarcinoma cells, or villous explant cultures unraveled that the coactivator promoted expression of genes associated with EVT identity, motility, and survival. Accordingly, depletion or chemical inhibition of TAZ, interacting with TEA domain family member 1 (TEAD1), impaired EVT differentiation, invasion, and migration and triggered apoptosis in the different trophoblast models. Notably, the coactivator also suppressed cell cycle genes and regulators of trophoblast self-renewal and prevented EVTs from cell fusion in organoids and primary cultures. Moreover, TAZ promoted human leukocyte antigen G (HLA-G) surface expression and increased NUAK1 kinase in EVTs thereby maintaining its own expression. In summary, the transcriptional coactivator TAZ plays a multifaceted role in the development of the EVT cell lineage by controlling different biological processes that initiate and preserve differentiation.

## Significance

A better knowledge of the developmental defects of placental trophoblasts is crucial for our understanding of several pregnancy diseases such as severe forms of fetal growth restriction and preeclampsia. Yet, molecular processes controlling early placental development have been poorly elucidated. The present study demonstrates that the HIPPO coactivator transcriptional coactivator with PDZ-binding motif (TAZ) plays a pivotal role in the differentiation program of invasive extravillous trophoblasts (EVT) that are crucial for immunological acceptance of the fetus and adaption of blood flow to the placenta. TAZ promoted EVT differentiation, migration, and survival and inhibited EVTs from undergoing cell fusion, the differentiation pathway of the hormone-producing syncytiotrophoblasts. Overall, TAZ controls EVT development by diverse mechanisms that trigger and sustain EVT differentiation.

Formation of the extravillous trophoblast (EVT) lineage of the human placenta and its different subtypes is crucial for early fetal development and progression of pregnancy (1–3). During the first weeks of gestation, EVTs originate from the trophoblastic shell, migrate into the uterine spiral arteries and plug these vessels to restrict maternal blood flow to the placenta and prevent oxidative damage of the tissue (4–7). At the time when matured placental structures develop, EVTs originate from specialized anchoring villi attaching to the maternal decidua (8, 9). Progenitors residing in the proliferative cell columns of these villi give rise to placental EVTs (pEVTs) that undergo endoreduplication upon differentiation in the distal regions of the columns (10). After detachment from anchoring villi, EVTs develop into endovascular EVTs (eEVTs) and into interstitial EVTs (iEVTs) that migrate into the uterine stroma where they encounter diverse maternal cell types (11, 12). Interaction of iEVTs with the different immune cells is required for immunological acceptance of the fetus and regulation of EVT cell function, whereas the combined action of eEVTs and iEVTs is necessary for remodeling of the spiral arteries (8, 13–17). The latter are transformed into low-resistance conduits allowing for low-pressure blood flow to the placenta and adapted hemotrophic nutrition of the fetus. Failures in the remodeling process are hallmarks of various pregnancy disorders including miscarriage, preterm labor, and severe forms of preeclampsia and fetal growth restriction (18–20). Inadequate immunological responses of the mother to paternal antigens as well as defects in the maturation of EVTs have been described (21–24). Yet, our present knowledge about the key drivers of EVT lineage commitment and differentiation during early placental development is still scarce.

Based on functional studies in different primary culture models as well as comparative analyses with other species a variety of signaling pathways and transcriptional regulators controlling trophoblast differentiation and invasion have been identified (1, 25–27). Expansion and survival of EVT precursors requires neurogenic locus notch homolog protein 1 (NOTCH1) that defines the progenitor niche of the cell column by suppressing markers of villous cytotrophoblast (vCTB) identity and self-renewal such as TEAD4 and ΔNp63 (28). However, formation of pEVTs in distal regions of the cell column is controlled by hypoxia, epithelial to mesenchymal transition, and transforming growth factor beta (TGF-β) signaling as well as by several key regulatory transcription factors driving differentiation such as *TC7L2*, *EPAS1*, *ASCL2*, *SNAI1*, *DLX3,* and *GCM1* (29–35).

Previous studies also suggested that HIPPO signaling plays a crucial role in human trophoblast development (36–40). The particular pathway regulates numerous biological processes such as organ size, tissue regeneration, stemness, cell fate decisions, differentiation, and survival (41–44). Accordingly, HIPPO signaling is controlled by a vast range of stimuli including biomechanical signals, metabolic cues, stress response as well as G-protein-coupled receptor activation (45, 46). Signaling through the latter results in phosphorylation of the coactivators of HIPPO signaling, Yes-associated protein (YAP), and transcriptional coactivator with PDZ-binding motif (TAZ) by the large tumor suppressor kinases 1/2 (LATS1/2) provoking their cytoplasmic retention or proteasomal degradation (HIPPO-On state) (42, 45). However when HIPPO is shut off, YAP and TAZ translocate to the nucleus and coactivate members of the TEA domain (TEAD) transcription factor family thereby promoting self-renewal and differentiation (47). In the human placenta, TEAD proteins and their coactivators likely play differential roles in these processes since their expression patterns vary between the distinct trophoblast subtypes. TEAD4 and YAP are the predominant factors in proliferative vCTBs, trophoblast stem cells (TSCs), and organoids and were shown to promote trophoblast self-renewal and expansion (36, 37). Moreover, YAP directly suppressed marker genes of the hormone-producing multinuclear syncytiotrophoblast (STB) (36). In contrast, TEAD1 and TEAD3 are abundantly expressed in EVT nuclei and genomic accessibility of their binding motifs is upregulated during EVT development (31, 36). Moreover, TEAD1 has been identified as a critical regulator of EVT differentiation in TSCs (48). While TAZ, also called WW domain containing transcription regulator 1 (WWTR1), seems to safeguard the TSC state in addition to YAP (39), its elevated expression in pEVTs suggests that the coactivator predominantly controls EVT cell function and differentiation (36). Indeed, the present study shows that TAZ plays a key regulatory role in EVT cell lineage development. By analyzing TAZ in differentiating TSCs, trophoblast organoids (TB-ORGs), primary EVTs, choriocarcinoma cells, and villous explant cultures, we herein show that the coactivator preserves EVT differentiation, migration, and survival, sustains EVT-specific gene expression as well as membrane-bound HLA-G, and maintains its own expression in an autocrine fashion. Moreover, TAZ determines the cell fate of EVTs by suppressing their differentiation into multinuclear cells.

## Results

### Nuclear TAZ Expression Is Associated with EVT Development.

**Fig. 1.**
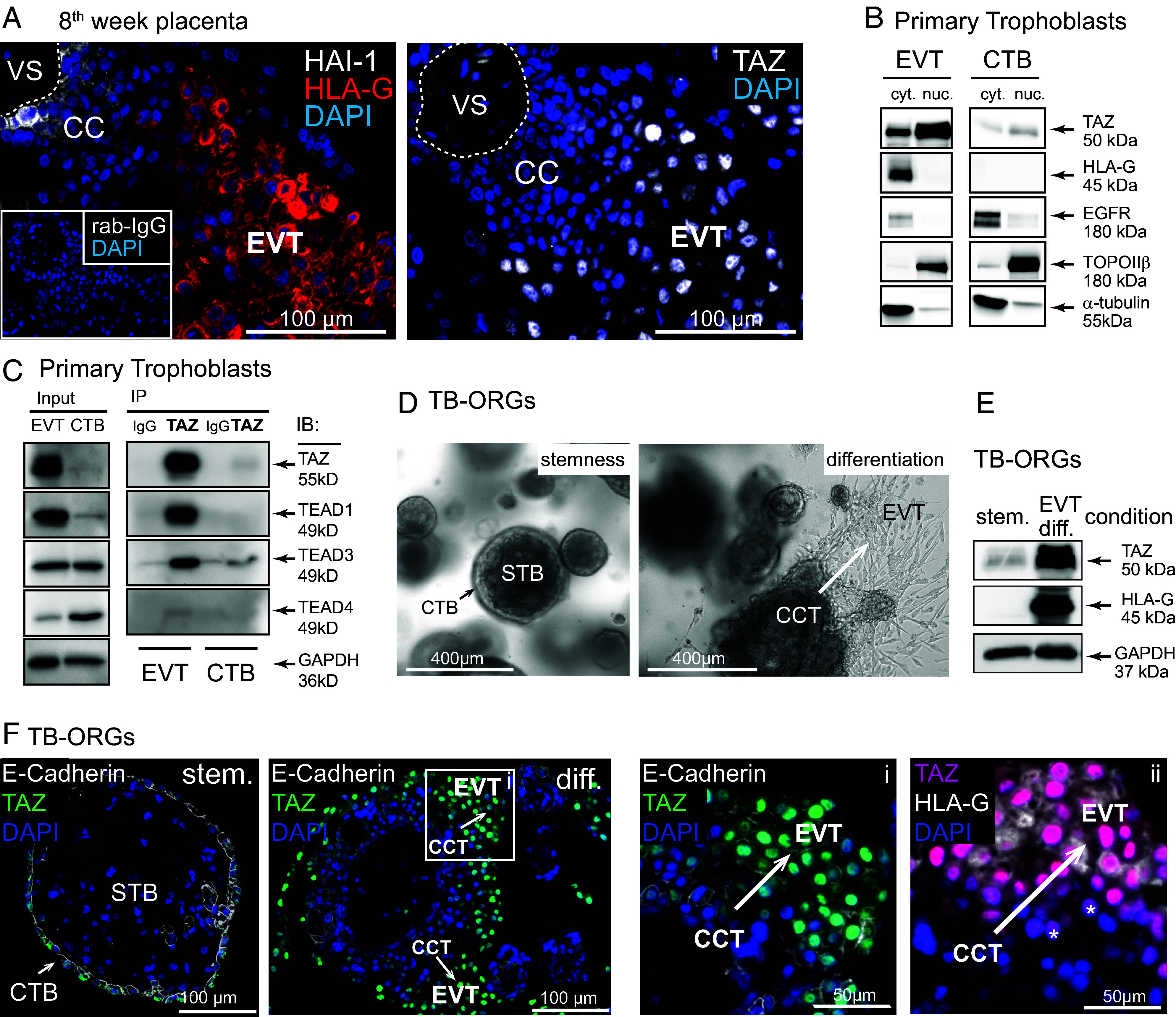
Expression pattern of TAZ in first-trimester placental tissues, primary trophoblasts, and TB-ORGs and its interaction with TEAD proteins. (*A*) TAZ IF in 8th week placenta. Representative serial sections of early placentae (6th to 9th week, n = 7) are shown. Inset picture depicts negative control staining using a primary rabbit IgG (rab-IgG). HAI-1 and HLA-G were used as markers of the proximal cell column (CC)/CTB and EVT, respectively. DAPI marks nuclei. The dashed line demarcates the villous stroma (VS). (*B*) Representative western blot showing cytoplasmic (cyt.) and nuclear (nuc.) distribution of TAZ in CTBs and HLA-G^+^ EVTs purified from first-trimester placenta (n = 3 different preparations, each isolated from 3 to 4 pooled 6th- to 9th-week tissues). Distribution of TOPOIIβ and α-tubulin indicate purity of nuclear and cytoplasmic extracts, respectively. (*C*) Representative coimmunoprecipitation of TAZ with TEAD proteins in CTB and EVT lysates of first-trimester placenta (n = 3, prepared from 4 to 5 pooled 6th- to 9th-week tissues). Immunoblots (IB) show expression of TAZ and TEADs in extracts before (input) and after immunoprecipitation (IP) with TAZ antibody or negative control IgG. GAPDH was used as a loading control for input extracts. (*D*) Light microscopy images of TB-ORGs (prepared from a single 6th-week placenta) grown under stemness or differentiation condition (absence of CHIR99021 for 10 d). (*E*) Representative western blot showing TAZ and HLA-G expression in lysates of self-renewing and differentiated TB-ORGs (derived from n = 3 single 6th- to 7th-week placentae). GAPDH was used as loading control. (*F*) Representative IF images detecting TAZ, E-cadherin, and HLA-G localization in sections of TB-ORGs (n = 3, prepared from single 6th- to 7th-week placentae) cultivated under stemness (stem.) and differentiation (diff.) condition. Inset picture (i) is shown at a higher magnification to the *Right*. (ii) is derived from the same region on a serial section. TAZ^+^/HLA-G^−^ EVTs are marked with stars. CCT, cell column trophoblast; CTB, cytotrophoblast; EVT, extravillous trophoblast; STB, syncytiotrophoblast.

The expression patterns of TAZ was investigated in early placental tissues, primary CTBs, and EVTs as well as in differentiating TB-ORGs derived from CTB progenitors (Fig. 1). Immunofluorescence (IF) in first-trimester placenta revealed that TAZ predominantly localized to nuclei of HLA-G^+^ pEVTs, whereas only faint signals were observed in the cytoplasm of some HAI-1^+^ EVT progenitors (Fig. 1*A*). Accordingly, the coactivator was abundant in nuclear lysates of isolated primary EVTs, while it was weakly expressed in CTB extracts (Fig. 1*B*). One of the potential binding partners of TAZ, TEAD1, was exclusively detected in EVT nuclei on placental tissue sections, whereas TEAD3 was present in both CTBs and STBs and enriched in EVTs (*SI Appendix*, Fig. S1*A*), as previously shown (36). Immunoprecipitation in primary cell lysates revealed that both TEAD1 and TEAD3 interacted with TAZ in EVTs, whereas the vCTB progenitor marker TEAD4, which disappears during EVT differentiation (36, 37), showed marginal binding (Fig. 1*C*). Furthermore, TB-ORGs, recapitulating cell column formation and EVT differentiation upon removal of the wingless (WNT) activator/GSK-3β inhibitor CHIR99021 from the stem cell medium (Fig. 1*D* and *SI Appendix*, Fig. S1 *B*–*D*), strongly upregulated TAZ in the newly developed HLA-G^+^ EVTs (Fig. 1 *E* and *F*). The latter also underwent polyploidization in vitro, reaching a 4N status (*SI Appendix*, Fig. S1 *E* and *F*), as recently demonstrated for pEVTs in vivo (10). Notably, nuclear TAZ expression preceded HLA-G expression during EVT formation in TB-ORGs and placental tissues (Fig. 1*F* and *SI Appendix*, Fig. S1*A*).

### TAZ Controls HLA-G Expression in Different Trophoblast Cell Models.

**Fig. 2.**
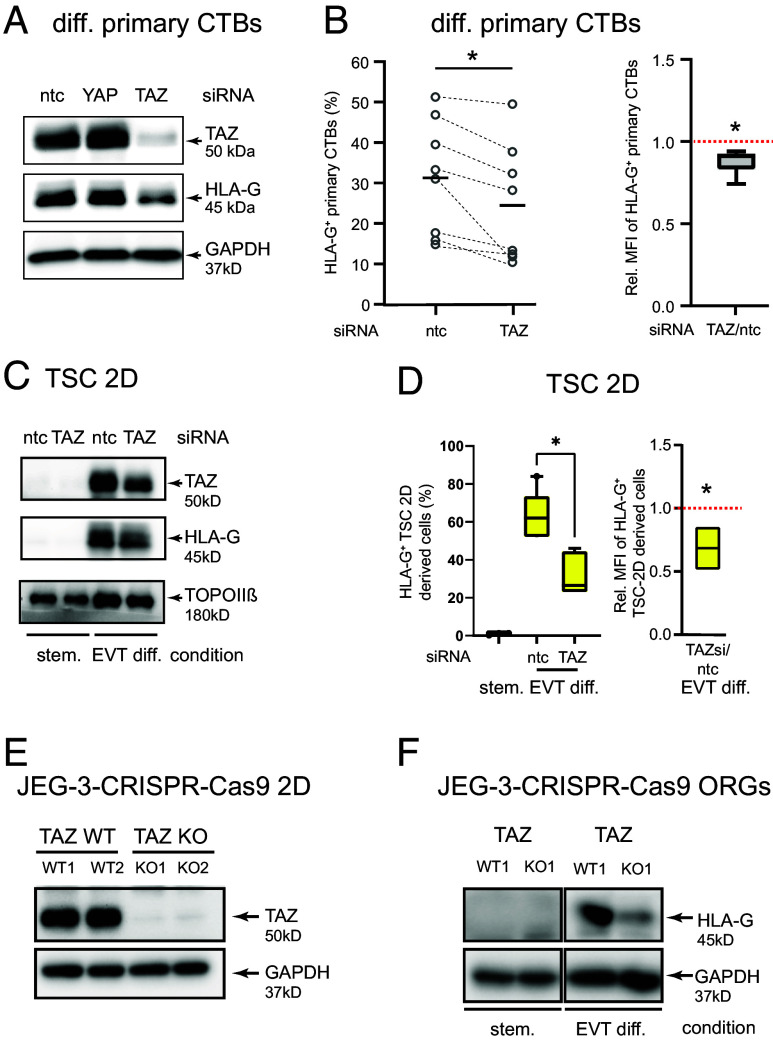
Genetic manipulation of TAZ in different trophoblast models affects HLA-G expression. (*A*) Representative western blot showing TAZ and HLA-G in protein lysates isolated from TAZ or YAP siRNA-treated primary CTBs after cultivation on fibronectin for 72 h (n = 5 different preparations, each isolated from 3 to 4 pooled 6th- to 9th-week placentae). GAPDH was used as a loading control. ntc, nontargeting control; (*B*) Flow cytometry analyses of TAZ siRNA-treated CTBs (n = 8, purified from 2 to 4 pooled 6th- to 9th-week tissues) cultivated for 72 h on fibronectin. Percentage of HLA-G^+^ cells (*Left* graph; values of each experiment connected with dashed lines) and the mean HLA-G fluorescence intensity (MFI) per cell (*Right* graph, box-whiskers plot; ratio TAZ siRNA/ntc) are shown. Median values are depicted. ntc, nontargeting control. *P < 0.05. (*C*) Immunodetection of TAZ and HLA-G in TAZ siRNA- or ntc-treated self-renewing (stem.) TSCs and TSCs differentiating into EVTs in 2D upon depletion (6 d) of the WNT activator CHIR99021 from the stem cell medium (EVT diff.). A representative western blot of n = 3 different cultures is shown. TOPOIIβ was used as loading control. (*D*) Flow cytometry analysis of TAZ siRNA-treated differentiating TSCs. Box whiskers blots show the percentage of HLA-G^+^ cells (*Left* graph) and their mean HLA-G fluorescence intensity (*Right* graph, ratio of TAZ siRNA/ntc) before (stem.) and after EVT differentiation of TSCs incubated with the different siRNAs. Median values of n = 3 different TSC preparations are depicted. *P < 0.05. (*E*) Representative immunoblot (n = 3) showing TAZ protein expression in the two JEG-3 CRISPR-Cas9 TAZ KO clones and the two WT clones cultivated in 2D. GAPDH was used as loading control. (*F*) Representative western blot (n = 3) depicting HLA-G expression in JEG-3 TAZ KO organoids cultivated under stemness conditions or in EVT differentiation medium (absence of CHIR99021 for 7 d). GAPDH was used as loading control.

To assess the role of TAZ in EVT maturation, the particular coactivator was genetically manipulated in differentiating primary CTBs, TSCs, and JEG-3 choriocarcinoma cells (Fig. 2). TAZ gene silencing in first-trimester CTBs, differentiating into EVTs on fibronectin, significantly decreased TAZ and HLA-G protein expression (Fig. 2*A* and *SI Appendix*, Fig. S2*A*). In contrast, small interfering ribonucleic acid (siRNA)-mediated downregulation of YAP in these cells did not affect HLA-G expression, but increased TAZ protein levels (*SI Appendix*, Fig. S2*A*), as previously shown (36). Furthermore, flow cytometry analyses revealed that TAZ gene silencing diminished the percentage of HLA-G^+^ EVTs in differentiating primary cultures and the mean fluorescence intensity of HLA-G per cell (Fig. 2*B*). In agreement with that, TAZ siRNAs also decreased TAZ protein expression, *WWTR1* and *HLA-G* transcript levels, total and membrane-bound HLA-G, as well as the number of HLA-G^+^ cells in TSCs undergoing EVT differentiation in the absence of CHIR99021 (Fig. 2 *C* and *D* and *SI Appendix*, Fig. S2 *B*–*E*). Like in TB-ORGs, EVTs generated in TSC cultures perform genome amplification. However, TAZ silencing did not affect their 4 N DNA content (*SI Appendix*, Fig. S2*F*). Stable TSC clones harboring a TAZ gene knock out (KO) could not be maintained, presumably due to the fact that the coactivator plays a role in TSC self-renewal (39) and trophoblast survival (see below). However, CRISPR-Cas9 gene editing in JEG-3 choriocarcinoma cells allowed establishing two different KO clones lacking TAZ RNA and protein (Fig. 2*E* and *SI Appendix*, Fig. S2*G*). JEG-3 cells form 3D organoids under stemness conditions and recapitulate aspects of EVT differentiation such as induction of HLA-G, ERBB2, and fibronectin upon removal of CHIR99021 from the culture medium (49). The JEG-3 TAZ KO clones built organoids with growth rates similar to wild type (WT) clones that increased in size during EVT differentiation (*SI Appendix*, Fig. S2*H*), as recently shown for JEG-3 cell organoids (49). Yet, induction of HLA-G in the TAZ KO cells was considerably downregulated (Fig. 2*F* and *SI Appendix*, Fig. S2*I*), again suggesting a crucial function of TAZ in EVT differentiation.

### Genetic Manipulation of TAZ Affects Genes and Pathways Associated with EVT Differentiation.

**Fig. 3.**
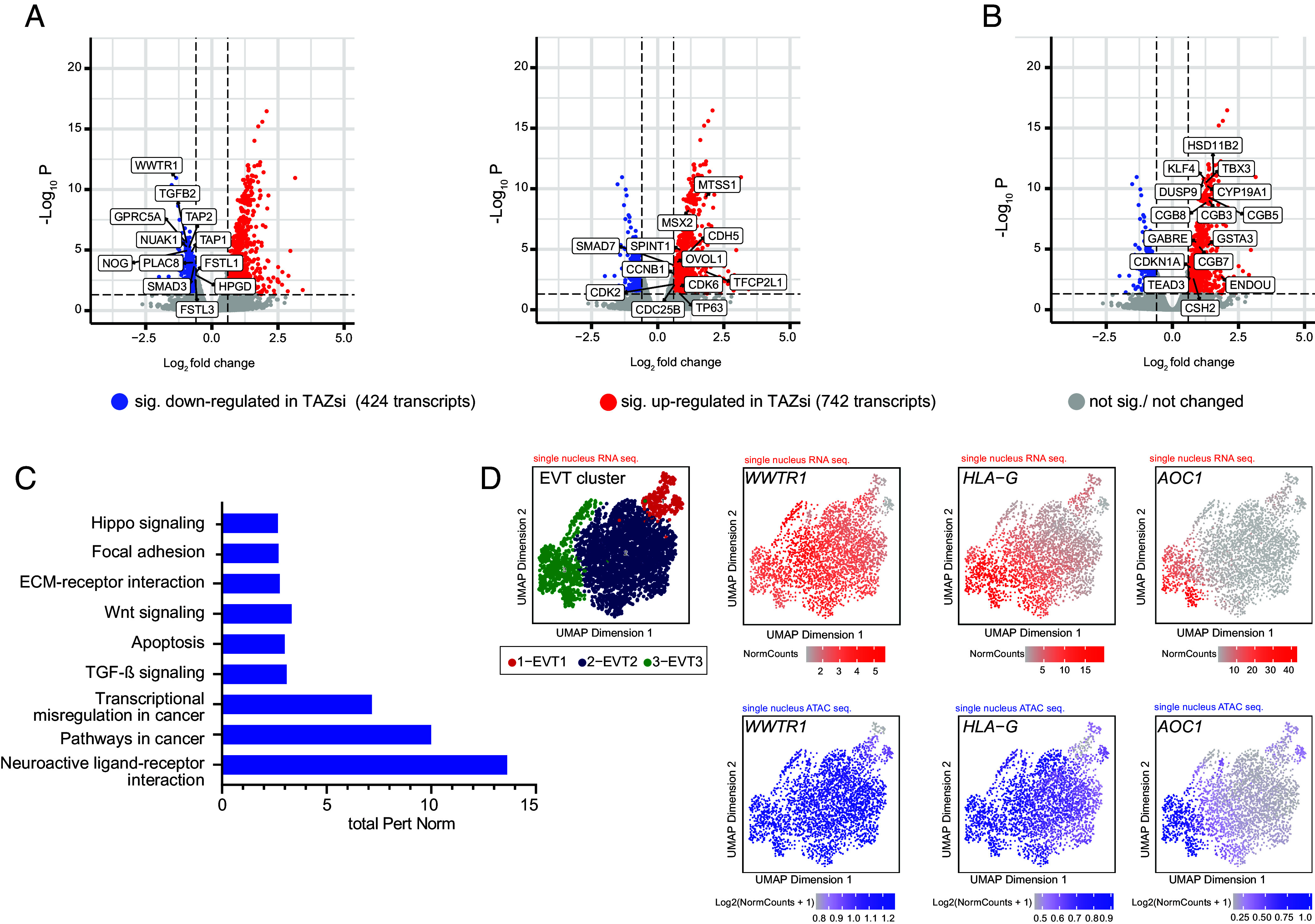
Bioinformatic analyses showing genes and pathways controlled by TAZ in different trophoblast cell models. (*A*–*C*) DEGs were evaluated by using bulk RNA-seq analyses of primary CTBs (n = 3, derived from 2 to 3 pooled 8th- to 10th-week placentae) after cultivation on fibronectin and treatment with TAZ siRNA or ntc for 72 h. (*A*) Volcano plots showing DEGs (dots illustrate individual transcripts, colored according to *P* values and log2 fold change (DESeq2, standard parameters, *P*adj < 0.05, DE: fold change > 1.5). *Left* graph shows a selection of EVT markers that were significantly downregulated by TAZ siRNAs, whereas *Right* graph highlights upregulated CTB progenitor markers and cell cycle genes. (*B*) Volcano plot depicting selected STB markers (DEGs) that were upregulated in TAZ-siRNA-treated primary CTBs. (*C*) Barplot of significantly affected KEGG pathways after gene silencing of TAZ in differentiating primary CTBs. Enrichment analysis was performed using the Pathway Express algorithm. (*D*) Uniform Manifold Approximation and Projection analyses of single-cell (sc) nuclear RNA-seq and sc nuclear ATAC-seq data showing expression and open chromatin of *WWTR1* and *HLA-G* in previously identified EVT clusters of first-trimester placentae (54). Please note that of all cell populations identified in the study of Ounadjela et al. only the EVT clusters are shown. The iEVT marker *AOC1*, encoding diamine oxidase (33, 55), is specifically expressed in EVT cluster 3 representing highly matured EVTs of the distal cell column. EVT clusters 1 and 2 depict EVT progenitors and differentiating pEVTs, respectively.

To capture TAZ-regulated genes during EVT development, primary CTBs, seeded on fibronectin, were treated with TAZ siRNAs for 72 h and subjected to bulk RNA-seq. Subsequent bioinformatic analyses revealed 1,148 differentially expressed genes (DEGs, 424 down-regulated and 742 up-regulated transcripts compared to nontargeting (ntc) controls (Fig. 3*A* and Dataset S1). Comparison with previously established gene signatures of purified first-trimester HLA-G^+^ and EGFR^+^ trophoblasts (50), indicated that 80 genes, diminished by TAZ-siRNAs, were enriched in the HLA-G^+^ cell pool (*SI Appendix*, Fig. S3*A*). The downregulated genes comprised EVT identity markers (*TEAD1*, *AOC1*, *NOTCH2*, *HLA*-*G*, *ITGA5*), regulators of cell migration and invasion (*PLAC8*, *GPRC5A*, *FSTL1* and *FSTL3*, *MMP2*, *MMP11*), TGF-β pathway genes (*TGFB2*, *SMAD3*, *HPGD, CTGF*), the BMP antagonist *NOG*, antigenic peptide transporters (*TAP1* and *TAP2*), required for HLA-G1 surface expression (51), and the kinases *NUAK1* and *NUAK2*, negatively affecting LATS activity (52, 53) (Fig. 3*A* and *SI Appendix*, Fig. S3*B*). Furthermore, TAZ gene silencing elevated CTB markers (*SPINT1*, *TP63*), trophoblast progenitor-specific transcripts (*CDH5*, *OVOL1*), cell cycle regulators (*CDK2*, *CCNB1*, *CDC25B*) as well as genes associated with trophoblast stemness and expansion (*MSX2*, *CDK6*, *TFCP2L1*) (Fig. 3*A* and *SI Appendix*, Fig. S3*B*). The raw RNA-seq data of TAZ siRNA-treated primary CTBs and controls are available at GEO database (GSE282830). Besides differentiating into EVTs, isolated CTBs, cultivated on fibronectin, also partly undergo spontaneous cell fusion. Despite its low abundance in vCTBs, TAZ knock-down was shown to increase STB formation in TSCs (39). Accordingly, silencing of the coactivator in primary CTBs elevated STB markers, hormone genes, and transcription factors associated with cell fusion (Fig. 3*B* and *SI Appendix*, Fig. S3*C*).

Bulk RNA-seq of the two JEG-3 TAZ KO and the two WT clones (accessible at GEO, GSE282831) unraveled 1,477 DEGs of which 102 mRNAs were downregulated in KO cells and elevated in the HLA-G^+^ EVT cell pool (*SI Appendix*, Fig. S3*D*). Comparison of DEGs of TAZ siRNA vs. ntc (primary cells) with DEGs of TAZ KO vs. WT revealed 206 common mRNAs (*SI Appendix*, Fig. S3*E*). Although many of the aforementioned DEGs of primary CTBs were either absent in JEG-3 cells or unchanged upon KO, several transcripts showed similar patterns of TAZ-dependent regulation between the siRNA-treated cultures and the JEG-3 KO clones (*SI Appendix*, Fig. S3 *B* and *C*). kyoto encyclopedia of genes and genomes (KEGG) analyses of the TAZ-silenced primary cells indicated significantly affected pathways such as HIPPO, WNT, and TGF-β signaling, apoptosis as well as migration- and cancer-associated routes (Fig. 3*C*). Moreover, gene concept network analyses suggested downregulation of extracellular matrix (ECM) and structure organization, BMP signaling, and regulation of actin filament-based processes in the TAZ siRNA-treated cells, whereas gene sets associated with carboxylic acid, sulfur, and anion transport were upregulated (*SI Appendix*, Fig. S3*F*). Furthermore, cluster analyses of previously published single-cell (sc) nuclear RNA-seq and sc nuclear assay for transposase-accessible chromatin with sequencing (ATAC-seq) data of first-trimester EVTs (54), suggested that *WWTR1* expression coincided with open chromatin of its EVT-specific target genes and their individual messenger ribonucleic acid (mRNA) expressions (Fig. 3*D* and *SI Appendix*, Fig. S3 *B* and *G*).

### TAZ Promotes Differentiation, Migration, and Survival of EVTs.

**Fig. 4.**
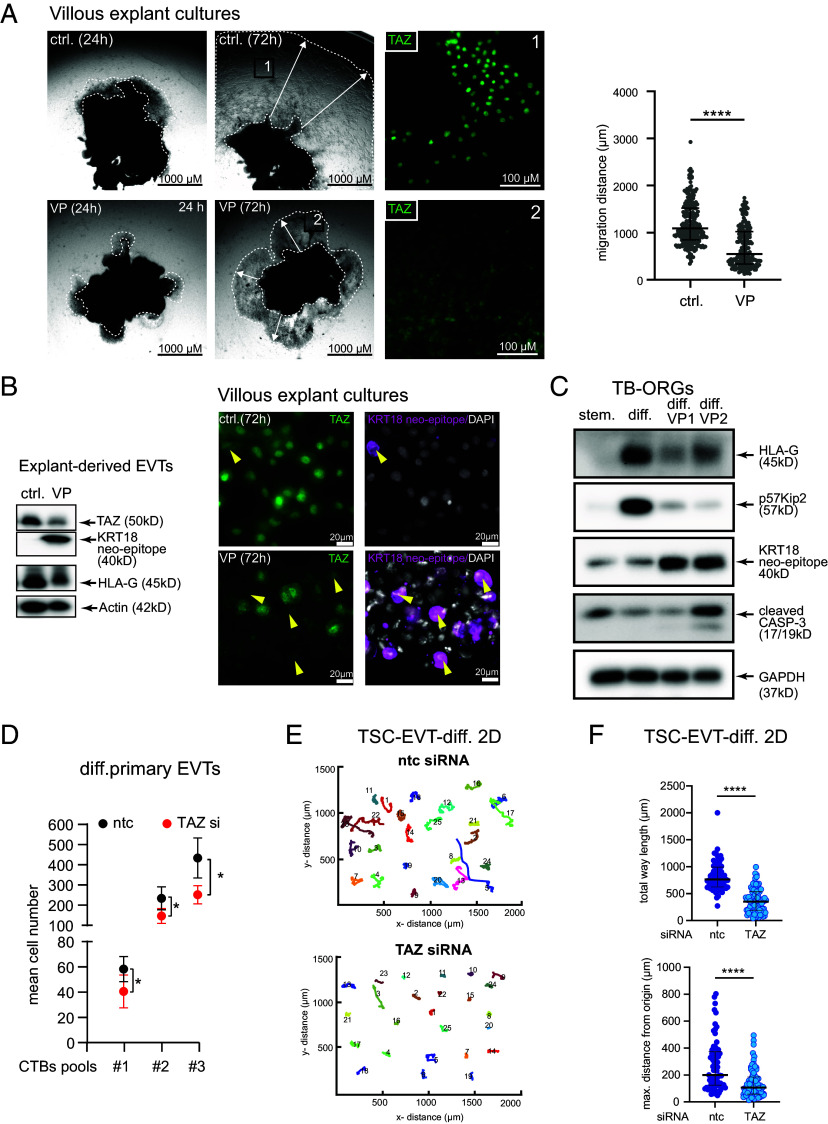
TAZ preserves EVT migration, differentiation, and survival. (*A*) Representative light microscopy pictures of villous explants seeded on collagen I for 24 and 72 h in the presence of VP. As control (ctrl.) Dimethyl sulfoxide (DMSO) was utilized. After whole-mount IF with TAZ antibodies two specific EVT areas of VP-treated and ctrl explants were photographed. Inset pictures with higher magnification depicting TAZ localization are shown to the *Right*. Scatter plot illustrates the migration distances of individual explants (ctrl., n = 27; VP, n = 24) derived from three first-trimester placentae (6th to 8th week). Each dot represents the distance between the outer rim of the migration zone and the position of the anchoring villus where EVTs detach from the column. Median values of 237 (ctrl.) and 187 (VP) measurements are shown. ****P < 0.0001. (*B*) Representative immunoblot (n = 3, left-hand side) depicting protein expression in lysates of EVTs, purified from villous explant cultures. Whole-mount IF pictures of explant cultures (n = 3) illustrating apoptosis in VP-treated EVTs (right-hand side). Arrowheads mark cells with high KRT18 neoepitope and low TAZ expression. (*C*) Representative western blot (n = 3) depicting expression of EVT- and apoptosis markers in self-renewing (stem.) and differentiating (diff.) TB-ORGs cultivated in the absence of presence of two different concentrations of VP (VP1: 0,14 µM, VP2: 0,28 µM). GAPDH was used as loading control. (*D*) Migration of primary EVTs through fibronectin-coated transwells. CTBs (n = 3, isolated from 3 to 4 pooled 6th- to 8th-week placentae), spontaneously differentiating into EVTs, were treated with TAZ siRNAs or ntc for 48 h before seeding on transwells. Mean values ± SD were obtained by counting five different KRT7 IF pictures, taken from the underside of membranes. Data of three individual experiments are depicted. *P < 0.05. (*E*) Cell tracking of TSCs that were differentiated into EVTs and incubated in parallel with ntc or TAZ-siRNAs for up to 6 d. 2D Migration was monitored for 48 h (between days 3 and 5 of differentiation) in each 25 single ntc and TAZ-siRNA-treated cells. A representative TSC experiment of n = 3 is shown. (*F*) Graphs show total way length and maximal distance from origin of 75 migratory TSCs derived from n = 3 cell tracking experiments. Median values are depicted. ****P < 0.0001.

To investigate role of TAZ in EVT migration, purified primary CTBs, differentiated EVTs, first-trimester villous explant cultures and TB-ORGs were treated with TAZ siRNAs or the chemical inhibitor verteporfin (VP), blocking the formation of YAP/TAZ–TEAD transcriptional complexes (Fig. 4). After 72 h a reduced migration distance in VP-treated explants, seeded on collagen I, as well as sequestration of the coactivator in the cytoplasm of outgrowing EVTs was noticed (Fig. 4*A*). In addition, the chemical inhibitor decreased TAZ and HLA-G protein levels in EVTs purified from these cultures, but induced KRT18 neoepitope suggesting that lowered TAZ concentrations also promoted apoptosis (Fig. 4*B*). Whole-mount IF stainings of the VP-treated explants revealed that, among the migration-restricted cells, EVTs with marginal TAZ protein levels showed high KRT18 neoepitope expression (Fig. 4*B*). Similarly, TAZ siRNAs decreased HLA-G expression in situ and induced apoptosis in EVTs of villous explants with either low or absent levels of the coactivator (*SI Appendix*, Fig. S4 *A* and *B*). Moreover, KRT18 neoepitope and cleaved caspase-3 were upregulated in lysates of TB-ORGs upon supplementation of VP, whereas the EVT markers HLA-G and p57KIP2 were downregulated (Fig. 4*C*). IF and flow cytometry of cells isolated from VP-treated TB-ORGs also revealed decreased TAZ levels and the characteristic apoptotic sub-2 N peak, respectively (*SI Appendix*, Fig. S4 *C* and *D*). Furthermore, TAZ siRNA treatment elevated KRT18 neoepitope expression in differentiating CTBs (*SI Appendix*, Fig. S4*E*) and decreased migration of primary EVTs through fibronectin-coated transwells (Fig. 4*D*). Finally, TSCs were differentiated into EVTs in the presence of TAZ siRNAs and their 2-dimensional (2D) motility was tracked using live cell imaging (Fig. 4*E* and *SI Appendix*, Fig. S4*F*). Gene silencing of the coactivator reduced migration of individual cells, their maximal distance from origin and total way length (Fig. 4 *E* and *F*). In the absence of CHIR99021, ntc-treated TSCs became spindle-shaped and highly migratory, whereas TAZ-siRNA-treated cells were less motile and displayed areas of cell fusion (*SI Appendix*, Fig. S4*G* and Movies S1 and S2).

### TAZ Inhibits Cell Fusion of EVTs.

**Fig. 5.**
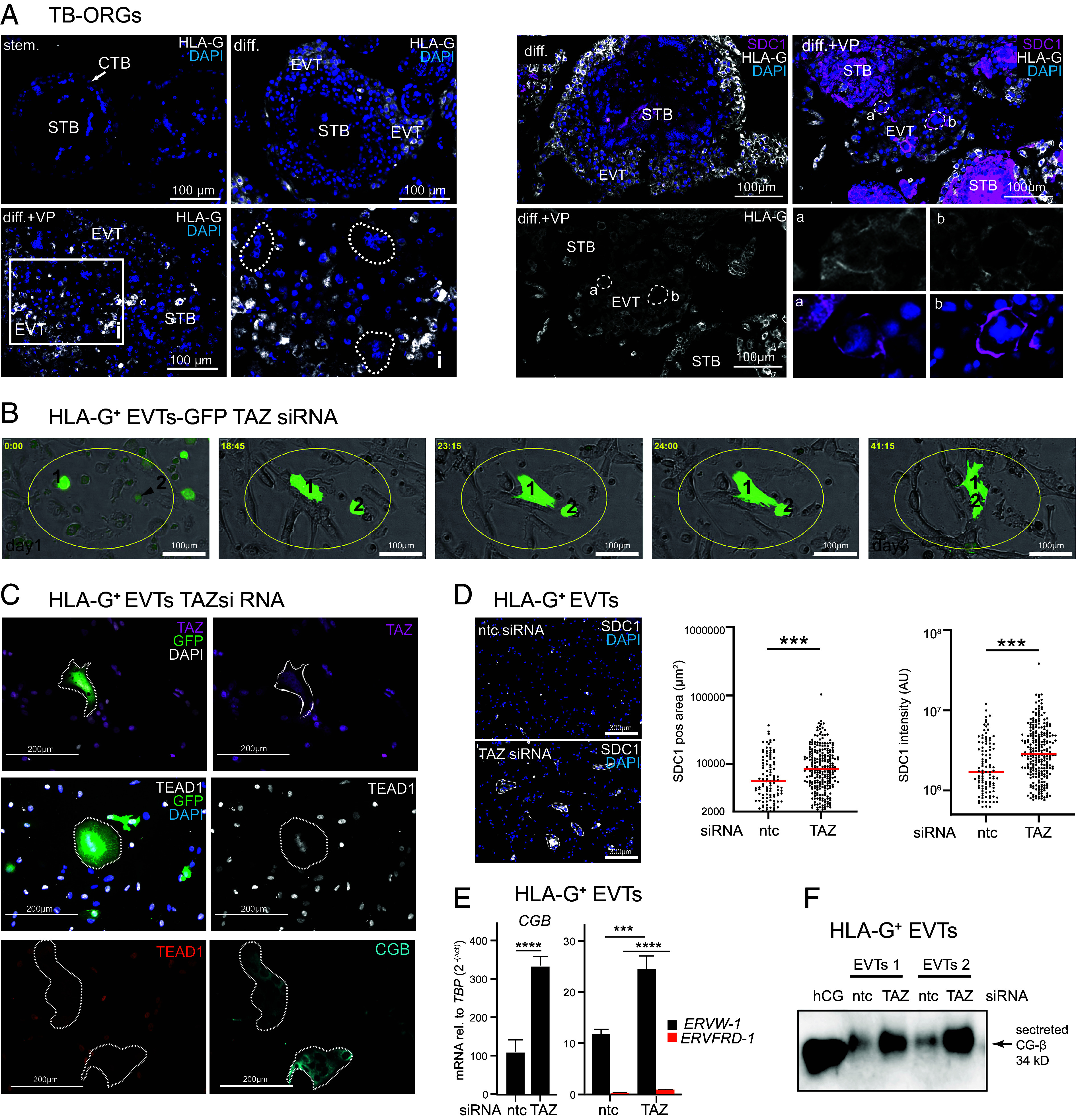
TAZ inhibits EVT cell fusion. (*A*) Representative IF pictures showing HLA-G (*Left* side) and SDC1/HLA-G (*Right* side) expression in TB-ORGs (n = 5, prepared from single 6th- to 8th-week placentae) grown under stemness (stem.) and EVT differentiation (diff.) condition in the absence or presence of VP. Directed EVT differentiation was performed as outlined in *SI Appendix*, Fig. S1*B*. Multinuclear cells within the HLA-G^+^ region are encircled by dashed lines. Inset picture (i, *Left* side) is shown at a higher magnification. Images on the *Right* side depict coexpression of HLA-G and SDC1 in selected multinucleated cells (a, b) of TB-ORGs. Magnified pictures (a and b) display single stainings of HLA-G and SDC1 demonstrating their colocalization at the membrane of the syncytial-like structures. DAPI marks nuclei. CTB, cytotrophoblast; EVT, extravillous trophoblast; STB, syncytiotrophoblast; (*B*) Selected microscopy images of HLA-G purified primary EVTs (n = 3, 6th- to 8th-week placenta) transfected with GFP plasmids and TAZ siRNAs for 72 h as outlined in *SI Appendix*, Fig. S5*E*. Pictures were taken between 24 and 72 h. (*C*) Representative IF images demonstrating multinucleated GFP^+^ cells coexpressing the EVT marker TEAD1 in TAZ siRNA-treated (72 h) HLA-G^+^ cultures. The TEAD1^+^ syncytial-like structures coexpressed CG-β. (*D*) IF showing SDC1 staining in ntc and TAZ siRNA-treated HLA-G^+^ EVTs (*Left* side). For quantification (n = 3 primary EVT preparations) SDC1 intensity and area of individual multinuclear structures, containing >2 nuclei per cell, were counted. Median values are shown ***P < 0.05. (*E*) qPCR showing *CGB*, *ERVW-1,* and *ERVFRD-1* transcript levels in HLA-G^+^ EVTs after incubation with ntc or TAZ siRNAs for 72 h. Mean values ± SEM of n = 5 cultures, measured in duplicates (normalized to *TBP*), are depicted. ****P < 0.0001; ***P < 0.0002. (*F*) Representative western blot (n = 4) showing secreted CG-β levels in ntc and TAZ siRNA-treated (72 h) primary EVTs. Urinary human CG (hu CG) was used as positive control.

The potential role of TAZ in inhibiting EVT cell fusion was investigated in differentiating TB-ORGs and HLA-G^+^-purified primary EVTs (Fig. 5). IF revealed that multinuclear, STB-like structures appeared in distal HLA-G^+^ regions of TB-ORGs upon treatment with VP (Fig. 5*A*). The multinucleated cells coexpressed markers of trophoblast cell fusion (SDC1, ENDOU) and HLA-G indicating an EVT origin (Fig. 5*A* and *SI Appendix*, Fig. S5*A*). Accordingly, the chemical YAP/TAZ inhibitor elevated *CGB* mRNA and protein expression in lysates of differentiating TB-ORGs (*SI Appendix*, Fig. S5 *B* and *C*). Moreover, VP also provoked induction of CG-β in individual mononuclear TEAD1^+^ EVTs (*SI Appendix*, Fig. S5*A*). To directly monitor EVT cell fusion in vitro, highly pure primary EVTs were transfected with green fluorescent protein (GFP) plasmids, incubated with ntc or TAZ siRNAs and analyzed using live cell imaging (*SI Appendix*, Fig. S5 *D* and *E*). Indeed, cell fusion of migratory GFP^+^ EVTs could only be observed upon TAZ gene silencing (Fig. 5*B*, *SI Appendix*, Fig. S5*F*, and Movies S3 and S4). The GFP-positive multinucleated cells coexpressed the EVT marker TEAD1, but lacked nuclear TAZ (Fig. 5*C*). In addition, fused TEAD1^+^ EVTs coexpressed CG-β (Fig. 5*C*). Compared to controls, the multinuclear cells of TAZ siRNA-treated EVT cultures also displayed an elevated SDC1^+^ area and intensity (Fig. 5*D*). Moreover, TAZ gene silencing in primary EVTs increased mRNA expression and secreted protein levels of CG-β as well as transcript levels of *ERVW-1* encoding the fusogenic protein syncytin 1 (Fig. 5 *E* and *F*). In contrast, *ERVFRD-1* (syncytin 2) was largely absent from EVTs (Fig. 5*E*), as previously shown (56). Differently to EVTs, GFP-transfected primary vCTBs showed little migratory behavior and formed large syncytial structures over time (*SI Appendix*, Fig. S5*F* and Movie S5).

### TAZ–TEAD1 Complexes Control Genes for EVT Migration, HLA-G Transport, and Autocrine TAZ Expression.

**Fig. 6.**
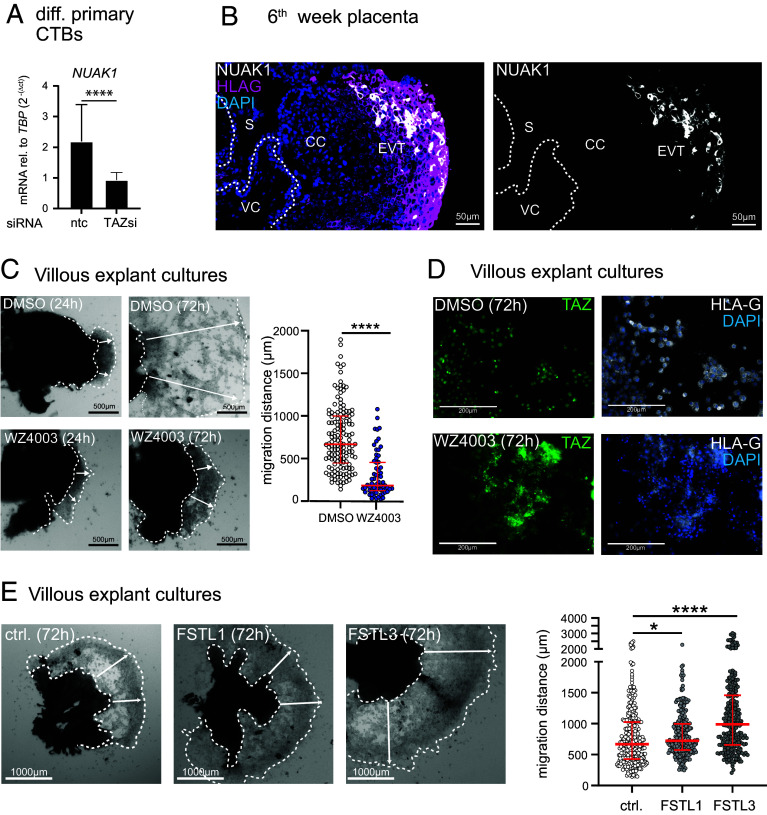
Identification of TEAD1–TAZ targets and their roles in TAZ expression and EVT migration. (*A*) qPCR showing *NUAK1* mRNA expression in differentiating primary CTBs after supplementation of ntc or TAZ siRNAs for 72 h. Mean values ± SEM of n = 5 cultures, measured in duplicates (normalized to *TBP*), are shown. ****P < 0.0001; (*B*) IF depicting NUAK1 expression, colocalizing with HLA-G^+^, in a subset of pEVTs. Representative images of early placentae (6th to 9th week, n = 3) are shown. DAPI marks nuclei. The dashed line encircles the villous core (VC). CC, cell column, S, syncytium. (*C*) Light microscopy images of villous explants, seeded on collagen I for 24 and 72 h in the absence (DMSO ctrl.) or presence of the NUAK inhibitor WZ4003. Scatter plot shows the migration distances (indicated by arrows) of explants at 72 h (DMSO, n = 21; WZ4003, n = 12) prepared from three first-trimester placentae (6th to 8th week). Median values of 145 (DMSO) and 66 (WZ4003) measurements are shown. ****P < 0.0001. (*D*) Representative whole-mount IF stainings (n = 3) illustrating the localization of TAZ and HLA-G in the DMSO and WZ4003-treated villous explants. (*E*) Light microscopy pictures of villous explant on collagen I, in the absence (ctrl.) or presence of 100 ng/mL human recombinant FSTL1 or FSTL3. Migration distances of cultures (ctrl, n = 36; FSTL1, n = 34; FSTL3, n = 34) prepared from three first-trimester placentae (6th week) were evaluated as mentioned above. Median values of scatter plots are based on different measures at 72 h (ctrl, n = 312; FSTL1, n = 249; FSTL3, n = 258). *P < 0.05, ****P < 0.0001.

TAZ–TEAD1 as well as TAZ–TEAD3 complexes could control EVT differentiation. Since *TEAD3* is less abundant than *TEAD1* and expressed in a lower number of EVTs (*SI Appendix*, Fig. S6*A*), we investigated binding of TEAD1–TAZ to the genomic regions of selected TAZ-activated genes. For this, promoter regions of *NUAK1*, *TAP1*, *TAP2, CTGF,* and *FSTL3* were analyzed by chromatin immunoprecipitation (ChIP)-qPCR (*SI Appendix*, Fig. S6 *B* and *C*). TEAD1 cognate sequences within these genes interacted with TEAD1 and TAZ when the chromatin of HLA-G^+^ EVTs was utilized (*SI Appendix*, Fig. S6*C*). In contrast, diminished genomic binding was observed in HLA-G^−^ primary CTBs. Transcript levels of *NUAK1*, a kinase that inhibits LATS activity (52, 53), decreased upon TAZ gene silencing in differentiating primary CTBs (Fig. 6*A* and *SI Appendix*, Fig. S3*B*). NUAK1 was specifically expressed in HLA-G^+^ EVTs in placental tissue, while NUAK2 was mainly detected in vCTBs and EVT progenitors (Fig. 6*B* and *SI Appendix*, Fig. S6*D*). Inhibition of NUAK with the chemical inhibitor WZ4003 impaired migration in villous explants and diminished nuclear localization of TAZ in these cultures (Fig. 6 *C* and *D*). Interestingly, analyses of previously published bulk RNA-seq data (33) revealed that *NUAK1* mRNA increased in TB-ORG-derived EVTs upon activation of TGF-β signaling, whereas expression of *NUAK2*, *TAP1*, *TAP2, FSTL1*, *FSTL3,* and *WWTR1* was not affected (*SI Appendix*, Fig. S6*E*). Finally, the ability of FSTL1 and FSTL3 to control trophoblast motility was analyzed in villous explant cultures. The respective recombinant proteins significantly elevated EVT migration, particularly FSTL3 (Fig. 6*E*).

## Discussion

Recent evidence suggested that the HIPPO signaling pathway plays a fundamental role in placentation. At the morula stage of mouse and human embryos TEAD4 and YAP are expressed in nuclei of outer blastomere cells and promote trophectoderm specification (57–59). At the postimplantation stage, TEAD4–YAP complexes are required for TSC self-renewal by promoting expression of stemness genes and cell cycle regulators (36, 37). The other crucial HIPPO coactivator, TAZ, has also been implicated in TSC expansion (39). However, YAP is the predominant HIPPO coactivator in proliferative trophoblasts and negatively controls TAZ expression. YAP KO or silencing elevated TAZ protein levels, whereas expression of dominant-positive YAP decreased the particular coactivator (*SI Appendix*, Fig. S2*A*) (36). YAP could promote GSK-3-mediated phosphorylation of TAZ on a unique N-terminal phosphodegron and its destruction in the proteasome as previously shown (60–62). Indeed, TAZ protein has a lower protein stability and higher turnover rate than YAP (60). Upregulation of TAZ upon loss of YAP could represent a compensatory mechanism to maintain trophoblast stemness and survival. Conversely, low TAZ concentrations in the presence of sufficient amounts of YAP could be crucial for maintenance of TSC self-renewal by preventing inadvertent differentiation of these cells into EVTs.

Indeed, the present study demonstrates that TAZ plays a multifaceted role in EVT development and differentiation. In contrast to proliferative CTBs, TAZ is abundantly expressed in EVTs in situ and predominantly shows nuclear localization. Accordingly, TSCs and TB-ORGs, undergoing directed EVT differentiation in vitro, considerably upregulated nuclear TAZ. Notably, TAZ protein concentrations increased much stronger than transcript levels during EVT differentiation of TSCs (Fig. 2*C* and *SI Appendix*, Fig. S2*C*), suggesting that protein stabilization largely dictates its abundance. Accordingly, faint cytoplasmic staining of the coactivator was noticed in EVT progenitors of the proximal CC, while their nuclei were negative (Fig. 1*A*). Interestingly, YAP is highly expressed in the proximal CC and might trigger the aforementioned TAZ degradation, while downregulation of YAP in EVTs, as previously observed in early placental tissues (36), could increase TAZ stability.

Herein, both TEAD1 and TEAD3 have been identified as TAZ binding partners in EVTs. However, *TEAD1* is more abundant than *TEAD3* and expressed in a larger number of EVTs (*SI Appendix*, Fig. S6*A*) (36). Moreover, TEAD1 protein is exclusively detected in EVTs, while TEAD3 is ubiquitously expressed among the different trophoblast subtypes (*SI Appendix*, Fig. S1*A*) (36). Hence, we speculated that TAZ–TEAD1 complexes, formed in EVTs, could be important drivers of the differentiation program of anchoring villi. Indeed, ChIP-qPCR analyses of the promoter regions of selected, EVT-specific TAZ target genes revealed occupancy of TEAD1 binding motifs with both TEAD1 and TAZ. Moreover, upregulation of TEAD1–TAZ seems to be an early event in EVT differentiation preceding the pEVT-specific expression of HLA-G in tissues and TB-ORGs (Fig. 1*F* and *SI Appendix*, Fig. S1*A*). Analyses of TAZ-siRNA-treated differentiating primary CTBs and TSCs as well as of JEG-3 organoids harboring a TAZ KO revealed that HLA-G expression was significantly compromised. While TAZ depletion downregulated *HLA-G* transcripts levels to various extents in the different cellular models (*SI Appendix*, Figs. S2*C* and S3*B*), decreased amounts of membrane-bound and/or total HLA-G was detected in all trophoblast systems. Diminished *HLA-G* expression could be an indirect consequence of an impaired differentiation process. However, TEAD1–TAZ transcriptional complexes binding and activating the *TAP* transporter genes, *TAP1* and *TAP2*, in EVTs could be required for effectively promoting surface expression of HLA-G. Indeed, TAP deficiencies were shown to be associated with decreased peptide loading and retention of HLA-G1 in the endoplasmic reticulum (51). Elevated expression of genes associated with CTB proliferation in TAZ gene-silenced primary cultures (Fig. 3*A* and *SI Appendix*, Fig. S3*B*) further supports the idea that the coactivator governs early steps of EVT development. At the stage when progenitors exit the mitotic cell cycle, the differentiating cells transiently express VE-cadherin (*CDH5*) in the middle region of the expanding CC (28). Increased levels of this gene upon loss of TAZ suggests that maturation into HLA-G^+^ pEVTs could be hampered. Similarly, upregulation of CTB markers, cell cycle genes, and transcriptional regulators of stem cell self-renewal might suggest that a proportion of TAZ-deficient cells could be stuck in the EVT progenitor state.

Besides driving early steps of EVT formation, TAZ could also promote subsequent stages of differentiation. The coactivator fostered expression of genes of the TGF-β pathway that plays a crucial role in the EVT maturation process. Compared to pEVTs, elevated expression of TGF-β receptors and SMAD3 was detected in iEVTs and TGF-β-dependent SMAD3 phosphorylation was required for the expression of iEVT-specific enzymes such as DAO (*AOC1*) and PAPPA2 (33). By promoting *TGFB2* and *SMAD3* expression TAZ might support autocrine TGF-β signaling in distally located pEVTs and prepare these cells for further maturation upon contact with decidua-derived TGF-β ligands. Notably, TGF-β has also been shown to activate nuclear SMAD2/3-TAZ complexes, that interact with TGF-β response elements (63, 64), thereby adding another layer of complexity to the versatile functions of the coactivator.

TAZ has been identified as an inducer of epithelial to mesenchymal transition promoting migration and invasion of various cancer cell types (65, 66). The present study demonstrates that the coactivator also plays a crucial role in trophoblast cell motility. TAZ gene silencing in primary CTBs differentiating into EVTs affected pathways associated with cell migration such as focal adhesion and ECM-receptor interaction, along with ECM and extracellular structure organization. Functional experiments further showed that chemical inhibition, disrupting TAZ–TEAD complexes, or TAZ gene silencing impaired migration in villous explant cultures and differentiating TSCs as well as invasion of primary EVTs. Accordingly, TAZ supported expression of genes that were shown to promote migration and/or invasion of trophoblastic HTR-8/SVneo cells (*PLAC8*, *FSTL3*) or different cancer cells (*GPRC5A*, *FSTL1*, *MMP11*) (67–71). The present study identified *FSTL3* as a direct target of TEAD1–TAZ complexes in HLA-G^+^ EVTs and revealed that both recombinant FSTL1 and FSTL3 elevated EVT migration. Moreover, TEAD1–TAZ also bound TEAD1 cognate sequences in the promoter region of *NUAK1* thereby promoting EVT-specific expression of this AMPK-related protein kinase. NUAK kinases were shown to phosphorylate LATS, which provokes inhibition of the latter and, as a consequence, accumulation of TAZ in the nucleus (52, 53, 72). Chemical inhibition of NUAK in villous explants resulted in cytoplasmic retention of TAZ and reduced EVT migration. Therefore, TAZ appears to sustain its nuclear expression in an autocrine manner by activating NUAK1 in distal regions of the anchoring villus. Interestingly, *NUAK1* has also been identified as TGF-β-SMAD-dependent gene in EVTs (*SI Appendix*, Fig. S6*D*) and other cells (72) strengthening the idea that a cross talk between HIPPO and TGF-β signaling could be crucial for EVT maturation and function.

While TAZ orchestrates a developmental program controlling differentiation and motility of EVTs, it also plays a crucial role in the survival of these cells. The majority of TAZ siRNA or VP-treated EVTs with reduced TAZ levels showed defects in migration, yet did not undergo apoptosis. However, EVTs with either absent or marginal nuclear TAZ expressed the apoptotic markers KRT18 neoepitope and cleaved caspase-3, suggesting that a threshold reduction in TAZ compromises EVT survival. Various genes could contribute to programmed cell death upon loss of the HIPPO coactivator. For example, TAZ was shown to inhibit the intrinsic apoptosis pathway by preventing the release of cytochrome C from mitochondria into the cytoplasm (73). The present study revealed that the expression levels of the mitochondrial proapoptotic BH3-domain proteins *BID*, *NOXA,* and *BOK* showed an upward trend upon TAZ gene silencing in differentiating EVTs, while *BCL2* was significantly elevated potentially balancing their apoptotic effects (Dataset S1).

The current study also highlighted that TAZ is necessary for maintaining the proper trajectory of differentiation and, consequently, EVT identity. VP treatment or TAZ gene silencing in TB-ORGs and purified HLA-G^+^ cells revealed that decreased TAZ levels provoked EVT cell fusion. The multinucleated areas coexpressed syncytial markers (SDC1, ENDOU, CG-β) and EVT-specific proteins (HLA-G, TEAD1) confirming their EVT origin. HLA-G expression gradually declined during EVT cell fusion (Fig. 5*A*), further supporting the crucial role of TAZ in maintaining EVT characteristics. Interestingly, VP induced the expression of CG-β and ENDOU in subset of mononuclear EVTs suggesting that these TAZ-inhibited cells could be prone to cell fusion (*SI Appendix* Fig. S5*A*). Formation of multinuclear EVTs could be triggered by the expression of the fusogenic protein syncytin-1 (*ERVW-1*), which was upregulated in TAZ-depleted, HLA-G^+^ EVTs. Hence, an autocrine hCG loop could foster EVT cell fusion since the hormone has been delineated as a regulator of STB formation promoting syncytin expression through the cAMP-dependent activation of GCM1 (74). Moreover, syncytialization has also been linked to the early phases of apoptosis involving initiator caspases (75). Therefore, reducing nuclear TAZ levels may sensitize EVTs to cell fusion by activating the initial stages of the intrinsic apoptosis pathway, while progressive loss of the coactivator causes programmed cell death.

**Fig. 7.**
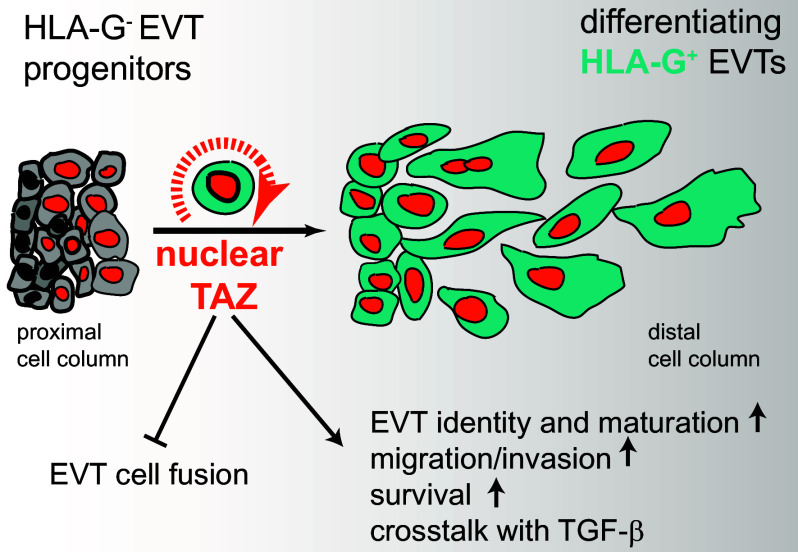
Schematic illustration of the role of TAZ in human EVT development. The coactivator promotes EVT differentiation, suppresses EVT cell fusion, and supports EVT migration, invasion, and survival.

In summary, the transcriptional coactivator TAZ plays multifaceted roles in human EVT development and differentiation (Fig. 7). It is required for the conversion of EVT progenitors into pEVTs and further maturation of the latter in conjunction with TGF-β signaling. Moreover, TAZ preserves the identity of EVTs by suppressing their capacity for cell fusion, safeguards survival, and promotes migration and invasion. The importance of TAZ in the anchoring villus is underscored by its autocrine regulation sustaining the HIPPO-Off state throughout EVT differentiation.

## Materials and Methods

### Tissue Collection.

Placental tissues (6th to 10th weeks of gestation) were collected from legal pregnancy terminations. Utilization of tissues and experimental procedures were approved by the ethical committee of the Medical University of Vienna (Nr. 084/2024). Written informed consent was obtained from women donating their placentae.

For the description of methods, see *SI Appendix, Methods*.

## Supplementary Material

Appendix 01 (PDF)

Dataset S01 (XLSX)

Movie S1. Migration of first trimester placenta-derived TSCs on fibronectin that were differentiated into EVTs and treated with non-targeting control siRNAs as outlined in *SI Appendix*, Fig. S4F. Live cell imaging was performed for 49 hours after day 3 of differentiation using Lionheart FX Automated Microscope.

Movie S2. Migration of first trimester placenta-derived TSCs on fibronectin that were differentiated into EVTs and treated with TAZ siRNAs as indicated in *SI Appendix*, Fig. S4F. Live cell imaging was performed for 49 hours after day 3 of differentiation using Lionheart FX Automated Microscope.

Movie S3. Live cell imaging of HLA-G^+^-purified first trimester primary EVTs on fibronectin. Cells were transfected with GFP plasmids and non-targeting control siRNAs as shown in *SI Appendix*, Fig. S5E. Imaging was performed for 44 hours using Lionheart FX Automated Microscope, one day after seeding and genetic manipulation.

Movie S4. Live cell imaging of HLA-G^+^-purified first trimester primary EVTs on fibronectin. Cells were transfected with GFP plasmids and TAZ siRNAs as depicted in *SI Appendix*, Fig. S5E. Imaging was performed for 44 hours using Lionheart FX Automated Microscope, one day after seeding and genetic manipulation.

Movie S5. Live cell imaging of purified first trimester villous CTBs on fibronectin. Cells were transfected with GFP plasmids as indicated in *SI Appendix*, Fig. S5E. Imaging was performed for 44 hours using Lionheart FX Automated Microscope, one day after seeding and GFP transfection.

## Data Availability

The RNA-seq data reported in this article have been deposited in the GEO database (accession no. GSE282830 and GSE282831) (76, 77).
