# Supplementary material for: The multifaceted roles of the transcriptional coactivator TAZ in extravillous trophoblast development of the human placenta
Source: Proc Natl Acad Sci U S A. 2025 Apr 14;122(16):e2426385122. doi: 10.1073/pnas.2426385122 (PMC12037006; doi:10.1073/pnas.2426385122)
Supplement: Supplementary file 1 — Appendix 01 (PDF) [file pnas.2426385122.sapp.pdf]

SI Appendix

The multifaceted roles of the transcriptional co-activator TAZ in extravillous trophoblast development of the human placenta

Gudrun Meinhardt[a], Hanna Waldhäusl[a], Andreas I. Lackner[b], Jasmin Wächter[a], Theresa Maxian[a], Anna-Lena Höbler[b], Sigrid Vondra[b], Victoria Kunihs[a], Leila Saleh[a], Peter Haslinger[b], Peter Kiraly[c], Andras Szilagyi[c], Nandor G. Than[c,d,e], Jürgen Pollheimer[b], Sandra Haider[a], Martin Knöfler[a,1]

[a]Placental Development Group, Reproductive Biology Unit, Department of Obstetrics and Gynaecology, Medical University of Vienna, Austria; [b]Maternal-Fetal Immunology Group, Reproductive Biology Unit, Department of Obstetrics and Gynaecology, Medical University of Vienna, Austria; [c]Systems Biology of Reproduction Lendulet Group, Institute of Enzymology, Research Centre for Natural Sciences, Budapest, Hungary; [d]Maternity Private Clinic of Obstetrics and Gynecology, Budapest, Hungary, [e]1st Department of Pathology and Experimental Cancer Research, Semmelweis University, Budapest, Hungary;

[1]Correspondence: Martin Knöfler, Department of Obstetrics and Gynaecology, Reproductive Biology Unit, Medical University of Vienna, Währinger Gürtel 18-20, 5Q, 1090 Vienna, Austria; e-mail: martin.knoefler@meduniwien.ac.at; phone: 0043-1-40400-28420.

SI Appendix Methods

Isolation and cultivation of primary trophoblasts. Primary cytotrophoblasts (CTBs) were isolated by enzymatic digestion of single, for organoid and stem cell cultures, or of pooled placentae between 6th and 10th week of gestation as described (1). Briefly, placentae were digested with three consecutive trypsin/DNAse I digestions and purified by Percoll gradient centrifugation. For spontaneous in vitro extravillous trophoblast (EVT) differentiation, immunoprecipitation (IP) and chromatin immunoprecipitation (ChIP) experiments, HLA-G-positive EVTs and HLA-G-negative CTBs were separated by using HLA-G PE MACS-positive sorting. CTBs or EVTs were seeded onto fibronectin-coated (20µg/ml Sigma) cell culture dishes and cultivated in DMEM Ham's F12 containing 10% FBS, 0.05 mg/ml gentamicin and 0.5µg/ml amphotericin B for 24 up to 72 hours. Supernatants were collected, cells were fixed for immunofluorescence staining, protein extracts were isolated for Western blotting and RNA samples were prepared for RNA-Seq and RT-qPCR. Isolation of cytoplasmic and nuclear extracts were performed according to the manufacturer´s instructions using the NE-PER extraction reagent (Pierce).

Cell culture of trophoblast organoids (TB-ORGs) and JEG-3 TAZ knockout (KO) and wild type (WT) organoids. Organoids were established by embedding CTBs (6-7th week, TB-ORGs) and JEG-3 cells (WT and TAZ KO) in growth factor-reduced Matrigel (Corning) domes. Cells were cultivated in organoid stem medium (Advanced DMEM/F12 supplemented with B27/1x ITS-X, 20µM L-glutamine, 10mM HEPES, 100ng/ml EGF, 1µM A-83-01, 3µM CHIR99021, 0.05 mg/ml gentamicin, 0.5µg/ml amphotericin B). For the formation of organoids 5 µM Y-27623 was added to the medium, which was omitted during further cultivation. For the directed formation of EVTs in TB-ORGs and JEG-3 organoids, cultures were grown in stem medium until the organoids reached a mean diameter of 150 µm. Organoids were then split and embedded again in growth factor-reduced Matrigel. Cell column formation and EVT development was induced by the withdrawal of CHIR99021 from the medium for 5 days. Subsequently, cultures were cultivated in organoid medium lacking EGF for another five days. In the TAZ inhibited conditions verteporfin (VP; 0.14-0.3 µM) was added. For the latter DMSO was used as control. Culture medium was changed every two to three days.

2D-Trophoblast stem cell (TSC) formation and EVT differentiation. For the establishment of TSC lines, primary CTBs collected from 6t^{h}-7th week placentae, were seeded onto fibronectin-coated dishes. Cells were cultivated in organoid stem medium containing 5µM Y-27623 (TSC medium). On the next day medium was changed and cells were cultivated until reaching 70% confluence. Cells were then split using TrypLE for up to 20 minutes and re-seeded. For the induction of EVT differentiation in 2D, TSCs were cultivated in TSC medium lacking CHIR99021 for three days or until cells reached confluence. Subsequently, cells were split again and further cultivated in TSC differentiation medium (absence of CHIR99021 and EGF). EVTs were collected after 3-4 days and further analyzed by flow cytometry,

immunofluorescence staining, Western blotting and qPCR. EVT differentiation was monitored by live cell imaging using a Lionheart FX (BioTek/ Agilent) device and Gen5 3.14 software. Pictures were taken every 45 minutes with 40x magnification.

Villous explant culture. Villous explant culture was performed as recently published (1). Briefly, villous tips were cut from 6th-8th week placental tissue and kept floating in cell culture medium (DMEM/F-12, 0.05mg/ml gentamicin, 0.5µg/ml amphotericin B) at 37°C/5% CO_2 overnight. On the next day explants were seeded onto collagen I domes. After 3 hours cultures were overlaid with medium (Advanced DMEM/F12, 20µM L-glutamine, 10mM HEPES, 0.05mg/ml, gentamicin 0.5µg/ml amphotericin) and further cultivated overnight allowing for cell column development. Next day the cultures were examined under the microscope, scored and divided into treatment groups containing explants with similar outgrowth rates. Explants were then treated with either FSTL1 (100 ng/ml), FSTL3 (100 ng/ml), WZ4003 (5 µM), VP (0.14-0.3 µM) or vehicle (0.1% BSA or DMSO) for up to 96 hours. EVT motility was monitored daily. The migration distance was assessed between 24-72 hours after treatment and was measured with Photoshop software CS5 (ruler tool). Floating explant cultures were cultivated with VP or DMSO, fixed and processed as mentioned in SI Appendix, Methods, Immunoflourescence staining of paraffin-embedded tissues. For the preparation of EVTs extracts from explants cultures, villous tissues were removed with forceps, the collagen dome containing EVTs was transferred into protein lysis buffer (Cell Signaling) and cells were homogenized using the Precellys Homogenizing Kit (CK14).

Gene silencing in differentiating CTBs, EVTs and TSCs. For the depletion of TAZ and YAP protein during the spontaneous differentiation of isolated CTBs and HLA-G$^+$ EVTs or TSCs, undergoing EVT differentiation after withdrawal of the GSK-3β inhibitor CHIR99021, cells were transfected with TAZ, YAP, or non-targeting control (ntc) siRNAs (ON-TARGETplus SMART pools, TAZ: L-016083-00-0005; YAP: L-012200-00-0005; ntc: D-001810-10-20; Thermo Scientific/ Dharmacon) one hour after seeding using Lipofectamine RNAimax (Invitrogen) according to the manufacturer´s instructions.

TAZ Gene Knock out. JEG-3 choriocarcinoma cells were transfected with the TAZ sgRNA (All-in-one sgRNA clones for human WWTR1 (HCP262223-CG01-3-B-d, HCP262223-CG01-3-B-e, HCP262223-CG01-3-B-f, GeneCopoeia) and Cas9 plasmids as well as the TAZ replacement plasmid (DC-HTN262223-D01-B, GeneCopeia) using DNAfectin Plus (ABM) as indicated by the manufacturer. The WT cell lines were established by transfection with an All- in one-sgRNA non-targeting control sgRNA plasmid (CCPTCR01-1-CG01, GeneCopoeia). Cells were cultivated for 24 hours in DMEM (Gibco)/10% FBS superior (Sigma S0615)/2mM L-glutamine (Gibco). Subsequently, cells were split and clones were selected by G418 (800 µg/ml for 24h) and puromycin (1 µg/ml) treatment. Single clones were picked and further cultivated in 96 well plates in the presence of puromycin. TAZ expression of clones was analyzed by qPCR and Western blotting.

GFP expression in primary HLA-G$^+$ EVTs/CTBs and live cell imaging. Up to 5 million HLA-G$^+$ primary EVTs were transfected with 2 µg pmaxGFP plasmid (Lonza) directly after isolation from placental tissues using the SG-Cell line Kit (Lonza) and the Amaxa 4D-Nucleofector (program EO-100) according to manufacturer´s instructions. After electroporation cells were incubated in RPMI medium for 10 minutes at 37°C in the incubator before seeding onto fibronectin-coated dishes. One hour later cells were treated with siRNAs. Next day medium was changed and cells were monitored with the Lionheart FX (BioTek/Agilent) live cell imaging microscope equipped with Gen5 3.14 software.

Immunofluorescence staining of cells. CTBs and EVTs were fixed in culture dishes using 4% paraformaldehyde (PFA) for 20 minutes at room temperature. Subsequently cells were washed twice with PBS, permeabilized with 0.5 % Triton X-100 for 10 minutes, and washed again. Unspecific binding was blocked with 5% FBS/0.3% Triton X-100/PBS for 30 minutes at room temperature according to the protocol of the manufacturer (Cell signaling). Cells were then incubated overnight at 4°C with primary antibodies (SI Appendix, Table S1), diluted in 1%BSA/PBS/0.3% Triton X-100. On the following day cells were washed and incubated with secondary antibodies (SI Appendix, Table S1) for 1 hour. Nuclei were counter stained with DAPI. All washing steps were performed with PBS. Slides were analyzed by fluorescence microscopy using the EVOS FL Imaging System or the Lionheart FX equipped with Gen5 software.

Immunofluorescence staining of paraffin-embedded tissues. Organoid cultures and placental tissues were fixed in 4-7.5% formaldehyde and subsequently embedded in paraffin. Tissue sections (3µm) were prepared and de-paraffinized with Xylol. After rehydration in descending ethanol concentrations the antigen retrival was performed in a KOS microwave Histostation (Milestone) using a citrate buffer at pH 6 (Sigma Aldrich #100369483). Unspecific antibody binding was blocked with 5% goat serum/TBS-T for 30 minutes at room temperature, primary antibodies (listed in SI Appendix, Table S1) were diluted in blocking buffer and incubated overnight at 4°C. On the next day slides were washed twice and incubated with secondary antibodies diluted in 1%BSA/TBS-T for one hour at room temperature. Nuclei were counterstained with DAPI and tissue sections were mounted using Fluoromount-G (SouthernBiotech Catnr. 0100-01). All washing steps were performed with TBS-T. Tissues were analyzed by fluorescence microscopy (Olympus BX53, cellSense-Standard software) and digitally photographed.

Whole mount staining of villous explant cultures. Collagen domes containing explant-derived EVTs were fixed with 4% PFA for 30 minutes. After washing with PBS (2x) cells were permeabilized with 0.5% Triton X-100 for 20 minutes, washed again and incubated for one hour with blocking buffer containing 5% FCS/0.3% Triton X-100/PBS at room temperature. Antibodies were diluted in 1% BSA/0.3% TritonX-100/PBS and applied over night at 4°C. After washing with PBS cells were then incubated with the secondary antibodies diluted 1:1000 and 1 µg/ml 4′,6-diamidino-2-phenylindole (DAPI, Roche) in 1%

BSA/0.3% TritonX-100/PBS for 1 hour at room temperature. The explants were removed with forceps and the collagen domes were transferred onto glass slides and mounted with Fluoromount-G.

Quantification of TAZ frequencies. The subcellular localization of endogenous TAZ along with DAPI was visualized by whole mount immunofluorescence staining of HLA-G-positive EVTs. The latter were derived from villous explant cultures, treated with TAZ, non targeting control (ntc) siRNAS for 72hours or the YAP/TAZ inhibitor VP (0.14 µM) for 48 hours. Pictures were taken with an Olympus BX53 microscope and cellSense-Standard software. The frequency of EVTs expressing nuclear TAZ was evaluated with Adobe PhotoshopCS5 software.

Evaluation of SDC1-positive areas in TAZ siRNA-treated EVTs. Equal cell numbers of HLA-G PE-sorted EVTs were seeded onto fibronectin-coated 48 well dishes (Nunc) and transfected with ntc/TAZ siRNAs 1 hour later. Cells were fixed at day 3, stained with SDC1 antibody (SI Appendix, Table S1) to visualize fused regions. Nuclei were stained with DAPI. SDC1-positive areas were photographed using a Lionheart FX device (BioTek/Agilent) equipped with Gen5 Image Prime 3.14 software. SDC1 positive areas were automatically measured using cellular analysis tool and intensities between ntc and TAZ siRNA-treated EVTs were compared. 9-12 pictures were taken per condition with a 40x magnification representing an area of approximately 22.5 to 38.8 mm^2. Only SDC1-positive areas larger than 2000 µm were taken into account representing areas with more than 2 cells.

Western blotting. Cell culture supernatants and protein extracts were separated on SDS/PAA- gels, transferred to ROTI PVDF-membranes (Carl Roth), incubated with primary antibodies (SI Appendix, Table S1), and diluted in 5% BSA/TBS-T overnight as previously described. On the following day membranes were washed and incubated with HRP-labeled secondary antibodies for one hour. For signal development and detection the WesternBright Chemiluminescence Substrat Quantum (BioZym) and the ChemiDoc imager (Bio-Rad) was used. Fiji/ImageJ was used for quantification of protein bands.

Reverse transcriptase-quantitative PCR (RT-qPCR). Total RNA was isolated with PeqGold Trifast (PeqLab) and reverse transcription was performed with RevertAid H Minus Reverse Transcriptase (Thermo Scientific). A 7500 Fast Real-time PCR system (Applied Biosystems), the Luna Universal Probe qPCR Master Mix (New England BioLabs) and the following TaqMan Gene Expression assays (ABI) were used according to manufacturer´s instructions: CGB3 (Hs00361224_gH), ERVFRD-1 (Hs01942443_s1), ERVW-1(Hs01926764_u1), HLA-G (Hs00365950_g1), NUAK1 (Hs00934234_m1), WWTR1 (Hs00210007_m1). Signals were normalized to TATA-box binding protein TBP (4333769F).

Flow cytometry of primary trophoblasts, TB-ORG-derived cells and 2D-differentiated TSCs. Primary trophoblasts were stained directly after isolation from placental tissues using the HLA-G PE antibody or a IgG PE control (SI Appendix, Table S1) for 30 minutes at 4°C in the dark. Cells were then washed twice with MACS Buffer (Miltenyi) and fixed using the BD Cytofix/Perm Kit (BD Biosciences) overnight. On the next day, cells were washed twice and incubated with 0.5% Triton X-100 for 5 minutes allowing permeabilization of the cell membranes. After washing with MACS buffer nuclei were stained with DAPI for 10 minutes. After two washing steps cells were re-suspended in MACS buffer, filtered through a 30 µm cell strainer and analyzed by flow cytometry (CytoFlex) and Flow-Jo software. TSCs and TSC-derived EVTs were collected by trypsinization with TrypLE for 10-20 minutes at 37°C. Cells were washed with cell culture medium, filtered, re-suspended in MACS buffer and stained as mentioned above. To harvest TB-ORGs-derived cells organoid domes were incubated with Cell recovery solution (Corning) for 60 minutes at 4°C. TB-ORGs were then collected by centrifugation and digested with TrypLE for 20 minutes at 37°C. For the preparation of single cell suspensions, TB-ORGs were vortexed during trypsinization and thoroughly pipetted up and down and filtered. Subsequently, cells were collected by centrifugation, washed again, re-suspended in MACS buffer, stained and analyzed as mentioned above.

EVT migration. CTBs isolated from pooled placentae were seeded onto fibronectin-coated 24 well dishes. One hour after seeding cells were washed with PBS to remove debris and STB- fragments and cells were transfected with ntc and TAZsi RNAs as mentioned above overnight. On the next day the cells were washed again. Subsequently, differentiating CTBs were collected by digestion with TrypLE after 48h in culture and seeded onto fibronectin-coated transwells (Millipore; 12 µm pore size) in duplicates per condition. After an additional 24 hours transwells were fixed with 4% PFA for 15 min at room temperature, washed with PBS, and the cells on the upper side of the membranes were removed with a cotton swab. Cells were permeabilized with 0.5% Triton X for 5 minutes, washed with PBS and stained with cytokeratin 7 and DAPI. Membranes were mounted onto glass slides and migrated CK7-positive cells were counted with image J/Fidji software. To track single cell migration of TSCs undergoing EVT differentiation in the presence of TAZsi or non-targeting control siRNAs, Celltracker software was used as described (2). Overall, the mobility of 75 cells (25 cells per condition, each derived from three TSC preparations) was evaluated for 48 hours between day 3 and day 5 of EVT differentiation. The total way length and maximal distance from the origin was measured.

Immunoprecipitation. Protein lysates from $2x10^6$ HLA-G^+ EVTs or CTBs were prepared according to manufacturer´s instructions (Cell signaling) and a TAZ antibody or rabbit IgG control (SI Appendix, Table S1) were used to precipitate protein complexes. The composition of the precipitated complexes was analyzed by Western blotting using antibodies (SI Appendix, Table S1) detecting members of the TEAD family of transcription factors.

Chromatin Immunoprecipitation (ChIP)-qPCR. For ChIP experiments the SimpleChIP Enzymatic Chromatin IP Kit with magnetic beads (Cell Signaling) was used. Briefly, chromatin from HLA-G^+ MACS-sorted EVTs and chromatin from the remaining HLA-G^- CTBs, isolated from 23 placentae (6th-10th week), was crosslinked with 1% formaldehyde for 10 minutes directly after isolation and further processed according to manufacturer´s instructions. After isolation of nuclei, enzymatic digestion and sonication, two pools of chromatin were established and two independent ChIP experiments were performed. Chromatin was incubated with TAZ and TEAD1 antibodies and rabbit IgG as negative control as listed in SI Appendix, Table S1. The immunoprecipitated chromatin fragments were isolated with ChIP grade magnetic beads, purified and further analyzed using a 7500 FAST Real-time PCR system (ABI) and the BrightGreen Express 2x qPCR MasterMix (ABM). Primer pairs amplifying regions next to the transcriptional start sites of *NUAK1*, *FSTL1*, *TAP1*, *TAP2*, and *CTGF* are listed in SI Appendix, Table S2. DNA binding of TAZ and TEAD was normalized to rabbit IgG control.

Prediction of binding sites in promoter regions. To analyze TAZ/TEAD1 bound regions the in promoters of selected target genes the GTRD database (3) was searched for TEAD4 and YAP1 chromatin immunoprecipitation datasets, as only one dataset was available for TAZ. These analyses revealed TEAD4 recognition motifs and/or TEAD4/YAP binding in the chromosomal loci of *NUAK1*, *FSTL3*, *TAP1*, *TAP2* and *CTGF*. The promoter regions of those genes were further searched for TEAD1 motifs using the Eukaryotic Promoter Database (EPD, (4, 5) and the Search Motif Tool using a p-value between 0.01 and 0.001 in regions between -5000 to -1000 bp and +100 to +1000 bp adjacent to the annotated transcriptional start sites (TSS). The promoter regions and annotated TEAD1 and YAP1 binding sites, retrieved from the GTRD database, were visualized using the Integrative Genomics Viewer (IGV version 2.18.10, Human (GRCH38/hg38) supporting transcription factor binding. The NCBI primer design tool Primer3 and BLAST was used to find suitable primer-pairs amplifying the regions of interest.

RNA-Seq. For RNA-seq experiments total RNA was isolated with the AllPrep DNA/RNA/miRNA Universal Kit (Qiagen) according to manufacturer´s instructions. The Core facility Genomics at the Medical University of Vienna prepared the sequencing libraries using the NEBNext Ultra II Directional RNA library Prep kit for Illumina (New England Biolabs). The quality of the libraries was checked using the High Sensitivity DNA Kit and a Bioanalyzer 2100 (Agilent). Pooled libraries were sequenced on a NextSeq500 instrument using 1x 75-bp single-end sequencing mode.

Bioinformatic analyses of RNA-seq data. Read qualities were checked with FastQC (6). Reads were mapped to the GRCh38.p13 human genome assembly and counted using STAR (version 2.7.5) (7). Principal components analysis was used to visualize samples and to detect potential outliers. For the TAZ siRNA-treated samples and their controls, 58 to 60 million reads (length=65) were processed, of which 88.4 to 91.5% were uniquely mapped to genomic regions. For the TAZ KO samples and their controls, 21 to 24 million reads (length=65) were processed, of which 88.3 to 90% were uniquely mapped

to genomic regions. Differential gene expression analysis was done using DESeq2 (version 1.22.0) (8). Differentially expressed genes were defined as those with an adjusted p-value < 0.05 and a fold change (FC) of ≥1.5. For HLA-G$^+$ vs. EGFR$^+$ trophoblast transcriptomic analysis, RNA-seq data were taken from GEO accession GSE126530, as published in our previous study (9). Venn diagrams and EnhancedVolcano plots were built by using BioVenn and EnhancedVolcano as described (10, 11). Functional enrichment was calculated and visualized in R (Bioconductor, enrichplot cnetplot; Gene-Concept Network) (12).

Pathway analysis. Significantly impacted pathways were identified using Pathway Express (13) as implemented in ROntoTools (version 2.14). The all-genes based analysis was selected, using the output from DESeq2, and analysis was carried out on the human pathways defined in the KEGG PATHWAY database (14). The total perturbation of a pathway (totalPert) and its adjusted p-value (pPert.fdr) were used to decide which pathways were significantly impacted.

Analyses of single nucleus (sn) RNA-seq and sn ATAC-seq data. The multiome data, including sn ATAC-seq and sn RNA-seq, were previously published and processed as described (15). Briefly, placental samples were collected from nine donors representing the first trimester of pregnancy (6th to 11th week). Sequencing reads were processed using Cellranger Arc on the Terra platform (16), followed by quality control and integration into ArchR projects for downstream analysis (17). For UMAP Visualization of EVT marker expression we focused on the EVT population annotated in the original dataset. UMAP embeddings for the EVT population were reanalyzed to visualize the expression of selected RNA- and ATAC-seq markers. Using ArchR, marker expression was projected onto the precomputed UMAP embeddings from the integrated multiome dataset. RNA-seq expression values were extracted from the gene expression matrix, and ATAC-seq gene scores were obtained directly from the ArchR project. All computational steps utilized the same QC and normalization methods described previously, ensuring consistency with the original analysis pipeline. All analyses and visualizations were conducted in R (v.4.2.3) using ArchR (v.1.0.3) and other standard bioinformatics packages. The computational workflow performed in this study is publicly available as part of the pipeline described in (15) [https://github.com/jian-shu-lab/hPlacenta-architecture]. Herein, we reused the same pipeline for different selected genes.

Statistical Analyses. Gaussian distribution was analysed with GraphPad Prism 10 using D'Agostino-Pearson normality test. Equality of variances was calculated with F test. Differences between two samples were analyzed with Student's t test, or Mann–Whitney U test. For the comparison of multiple groups one-way ANOVA and appropriate post hoc tests or Kruskal-Wallis or Friedman test were applied. A p-value of ≤0.05 was considered as statistically significant.

SI Appendix Tables

Antibody	Manufacturer	Cat.#	Species	Application
**TAZ/ WWTR1**	Sigma	HPA007415	Rabbit	WB 1:1000; IF-P / IF 1:200
**TAZ (D3I6D)**	Cell Signaling	70148	Rabbit	ChIP 1:50; IP 1:50
**TEF-1 (E-5)= TEAD1**	Santa Cruz Biotechnology	sc-393976	Mouse	WB 1:1000; IF-P 1:100
**TEAD1 (D9X2L)**	Cell Signaling	12292S	Rabbit	ChIP 1:50
**TEAD3**	GeneTex	GTX32917	Rabbit	WB 1:1000; IF-P 1:300
**TEAD4**	Sigma	HPA056896	Rabbit	WB 1:1000; IF-P 1:200
**TEF-3 = TEAD4**	Santa Cruz Biotechnology	sc-101184	Mouse	WB 1:1000
**HAI-1 (H180)**	Santa Cruz Biotechnology	sc-30205	Rabbit	IF-P 1:100
**HLA-G (4H84)**	Santa Cruz Biotechnology	sc-21799	Mouse	WB 1:1000; IF-P / IF 1:200
**EGFR (D38B1)XP**	Cell signaling	4267	Rabbit	WB 1:1000
**PAGE4**	Sigma	HPA023880	Rabbit	IF-P 1:500
**E-cadherin**	BD Transduction Lab	610181	Mouse	IF-P 1:200
**KRT18 neo-epitope (M30 CytoDEATH)**	Roche	12140322001	Mouse	WB 1:500; IF-P 1:50; IF 1:50
**p57 Kip2**	Cell Signaling	2557	Rabbit	WB 1:1000
**Cleaved Caspase-3 (Asp175) (5A1E)**	Cell Signaling	9664	Rabbit	WB 1:1000
**SDC1**	Sigma Aldrich	HPA006185	Rabbit	IF-P 1:250; IF 1:250
**CG-β**	Dako	A0231	Rabbit	WB 1:1000; IF-P 1:300; IF 1:300
**ENDOU**	Sigma Aldrich	HPA012388	Rabbit	IF-P 1:250
**NUAK1 (ARK5)**	Cell Signaling	4458S	Rabbit	IF-P 1:500
**KRT7**	Dako	M7018	Mouse	IF 1:200
**Actin**	Sigma	A2066	Rabbit	WB 1:2500
**α-tubulin**	Calbiochem	CP06	Mouse	WB 1:5000
**GAPDH (14C10)**	Cell Signalling	2118	Rabbit	WB 1:5000
**TOPO- IIβ**	BD Transduction Lab.	611492	Mouse	WB 1:1000
**HLA-G-PE (MEM-G/9)**	Exbio	1P-292-C100	Mouse	Cell sorting (MACS) 1:20; FC 1:400
**PE Mouse IgG1 isotype ctrl. (MOPC-21)**	Biolegend	400114	Mouse	FC 1:2000
**Normal rabbit IgG**	Cell Signaling	2729		ChIP 1:1000
**Rabbit IgG isotype ctrl.**	Cell Signaling	3900	Rabbit	IF-P, IF
**Mouse IgG isotype ctrl.**	Exbio	11-457-C100	Mouse	IF-P, IF
**Anti-mouse 488**	Invitrogen	A11017	Goat	IF-P / IF 1:1000
**Anti-rabbit 488**	Invitrogen	A11070	Goat	IF-P / IF 1:1000
**Anti-mouse 568**	Invitrogen	A11019	Goat	IF-P / IF 1:1000
**Anti-rabbit 568**	Invitrogen	A21069	Goat	IF-P / IF 1:1000
**Anti-mouse, HRP**	Cell signaling	7076	Horse	WB 1:10000
**Anti-rabbit, HRP**	Cell signaling	7074	Goat	WB 1:10000
**Mouse Anti-rabbit IgG (Light chain spec.)**	Cell signaling	45262	Mouse	WB 1:2000

Table S1. Primary and secondary antibodies. IF, Immunofluorescence, IF-P, immunofluorescence of paraffin sections; WB: Western blotting; ChIP, chromatin immunoprecipitation; IP, immunoprecipitation

Gene Symbol	Promoter ID (EPD)	Primer name	Sequence (5´-> 3´)	Primer position in the genome (GRCh38/hg38)	Amplicon [bp]
**CTGF**	**CTGF_1**	CTGF-F	TTCTGTGAGCTGGAGTGTGC	chr6:131951558-131951578	153
		CTGF-R	GCCAATGAGCTGAATGGAGT	chr6:131951426-131951445	
**FSTL3**	**FSTL3_1**	FSTL3site1_F	CACCCACGGCTGCTATAACA	chr19:672312-672331	231
		FSTL3site1_R	TAAGTGGGATTACAGGCGCC	chr19:672523-672543	
		FSTL3site2_F	ACTAAGGCAGAACTTGGCCC	chr19:674927-674946	112
		FSTL3site2_R	CCTGAGTGAGATCTGTGCCC	chr19:675020-675039	
**NUAK1**	**NUAK1_1**	NUAK1-F	CCCTCGCAGGCACTCATTTA	chr12:106138073-106138092	106
		NUAK1-R	AAAACACCGGACTTGAGGGG	chr12:106137986-106138006	
**TAP1**	**TAP1_1**	TAP1-F	CAGATCTGCCCCGAGACAAG	chr6:32854052-32854071	119
		TAP1-R	TTTCTCCATCACGCACACCC	chr6:32853953-32853971	
**TAP2**	**TAP2_1**	TAP2_1-F	GAGACTCATTGCAGGCCTGT	chr6:32838899-32838919	206
		TAP2_1-R	GTCTCTCCCAACCTCGCTAC	chr6:32838712-32838732	
	**TAP2_2**	TAP2_2-F	GTCTTGATGTGGAGGCTGCT	chr6:32837809-32837828	121
		TAP2_2-R	ACCTCCAACTCACAACGTCC	chr6:32837707-32837726	

Table S2. Primers used in ChIP-qPCR. Two different regions (*FSTL3* site1 and site 2) of the *FSTL3_1* promoter were amplified. In the *TAP2* gene two alternative promoters *TAP2_1* and *TAP2_2* were analysed. EPD, Eukaryotic Promoter Database; GRCh38, Genome Reference Consortium Human Build 38; chr, chromosome; bp, base pairs;

SI Appendix Figures

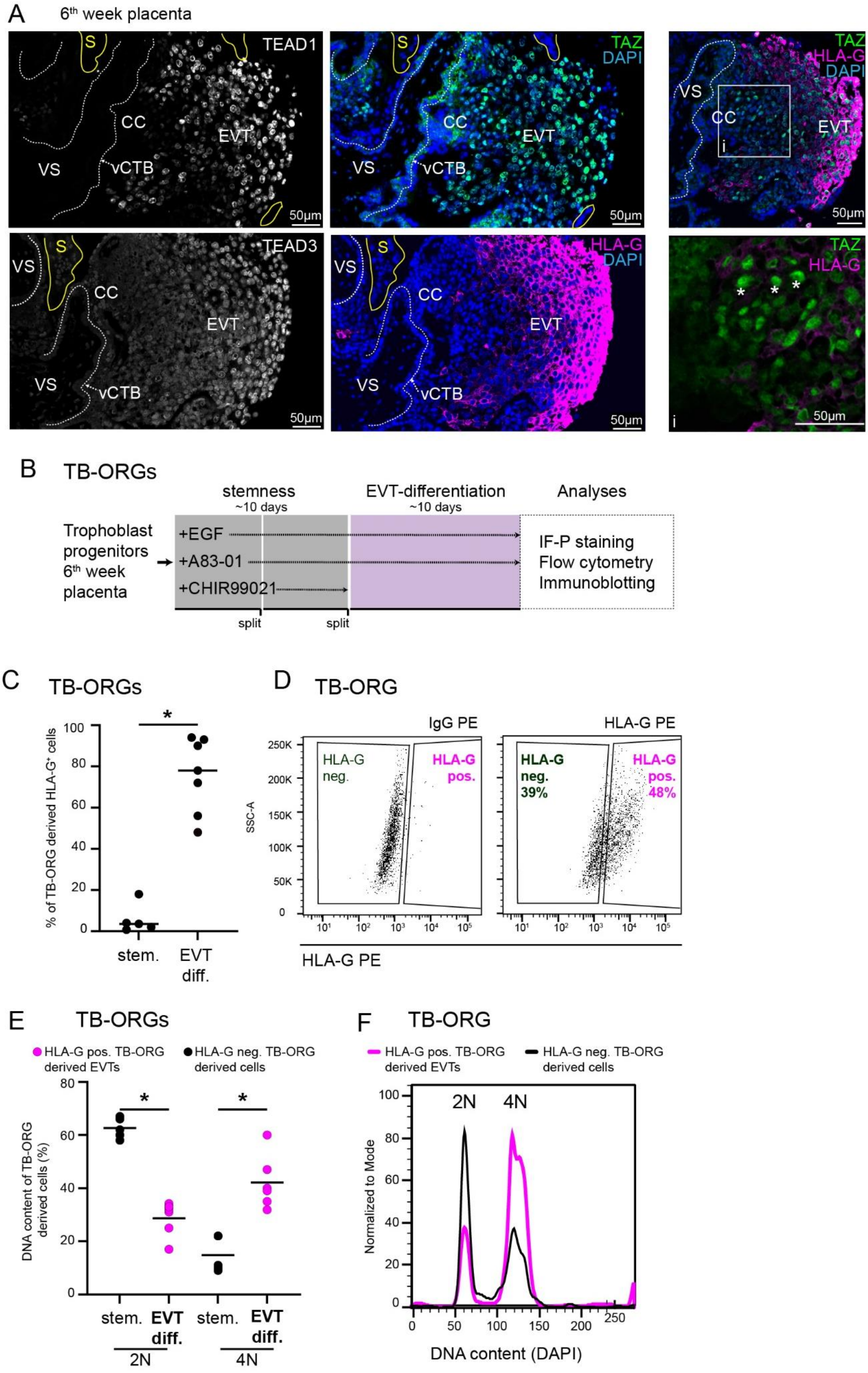

Fig. S1. TEAD expression in early placenta and HLA-G expression and polyploidization in differentiating TB-ORGs. (A) Representative immunofluorescence (IF) showing TEAD1, TEAD3, TAZ and HLA-G localization in 6th week placental tissues. Nuclei are stained with DAPI. Picture to the top right shows TAZ and HLA-G co-staining, inset picture (i) with a higher magnification is shown below. $TAZ^+/HLA\text{-}G^-$ EVTs are marked with stars. CC, cell column; EVT, extravillous trophoblast; vCTB, villous cytotrophoblast (dashed lines); VS, villous stroma; S, syncytium (marked by yellow lines); (B) Schematic presentation of the conditions used for self-renewal and EVT differentiation in TB-ORGs. For differentiation, CHIR99021 was removed after passage 2. (C) Percentage of $HLA\text{-}G^+$ cells in TB-ORGs, cultivated under stemness (stem., n=5, prepared from single 6th to 8th week placentae), and EVT differentiation condition (EVT diff., n=7, prepared from single 6th to 8th week placentae, 10 days in the absence of CHIR99021). HLA-G surface expression was analysed by flow cytometry. Median values are depicted. *, $p<0.05$ (D) Representative scatter plot showing distribution of $HLA\text{-}G^+$ and $HLA\text{-}G^-$ cells in a single TB-ORG culture at day 10 of EVT differentiation. IgG PE was used as a negative control. (E) DNA content in $HLA\text{-}G^-$ and $HLA\text{-}G^+$ cells isolated from TB-ORGs (prepared from single 6th to 8th week placentae) before (stem., n=5) and after EVT differentiation (n=7). DNA content and HLA-G expression were analysed by flow cytometry after staining with DAPI and HLA-G PE, respectively. Mean values are shown. *, $p<0.05$ (F) Representative flow cytometry histogram showing DNA content in a single TB-ORG preparation at day 10 of EVT differentiation.

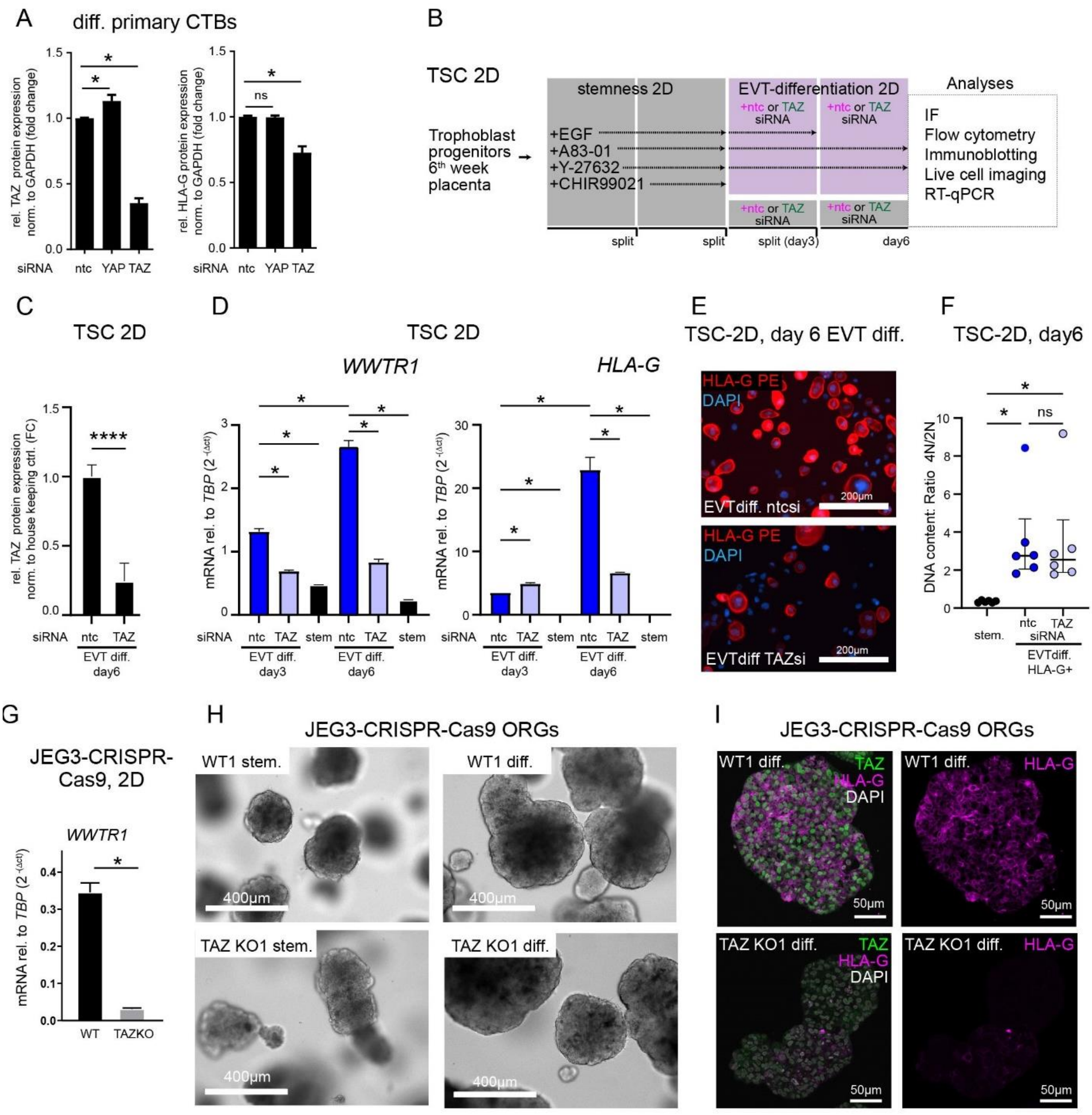

Fig. S2. HLA-G and TAZ expression in TAZ siRNA-treated primary CTBs/TSCs and TAZ knock out clones. (A) Quantification of TAZ and HLA-G protein expression after differentiation (72 hours on fibronectin) of TAZ siRNA- and non-targeting control (ntc)-treated primary CTB isolates derived from 3-4 pooled 6th to 9th week placentae (n=3). Protein levels were normalized to GAPDH. Mean values ± SEM are shown. *, p<0.05, ns, not significant. (B) Schematic illustration of culture conditions used for 2D TSC self-renewal and EVT differentiation. Treatment with siRNAs was started at passage 2. (C) Quantification of TAZ protein expression in TSCs after six days of EVT differentiation, normalized to house keeping control (ctrl.). Mean values ± S.D. of seven independent experiments are depicted. ****, p<0.0001; (D) Quantification of *WWTR1* and *HLA-G* transcript levels in self-renewing TSC cultures (stem.) and at day three and six of EVT differentiation using qPCR. Mean values ± SEM (normalized to *TBP*) of each n=2 siRNA-treated TSC cultures, measured in duplicates, are depicted. *, p<0.05. (E) Representative immunofluorescence pictures showing HLA-G expression in isolated TAZ siRNA and

ntc-treated TSCs at day 6 of EVT differentiation, prior to flow cytometry analysis. (F) DNA content (DAPI signals) of HLA-G^+ cells in self-renewing and differentiated TSCs (n=6) after incubation with TAZ siRNA or ntc. Median values (ratio 4N/2N) are shown. *,p<0.05, ns, not significant. (G) Quantification of *WWTR1* mRNA (qPCR) in the two JEG-3 TAZ KO and WT clones (prepared in replicates) grown under standard culture conditions in 2D. Mean values ± SEM (normalized to *TBP*) of n=2 different experiments, measured in duplicates, are shown. *, p<0.05. (H) Representative light microscopy images of JEG-3 WT and TAZ KO clones grown as organoids in 3D under stemness conditions or in differentiation medium (absence of CHIR99021) for seven days. (I) Representative immunofluorescence co-stainings detecting TAZ and HLA-G in sections prepared from JEG-3 WT and TAZ KO organoids at day 7 of EVT differentiation. DAPI marks nuclei.

A

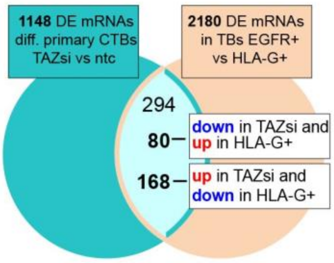

B

EVT marker	down in		up in HLAG+
Gene name	CTBs TAZsi	JEG-3 TAZ KO	TBs HLA-G+ vs EGFR+
TAZ/WWTR1	-2.5	-9.5	+3.9
CTGF/CCN2	-2.1	+3.8	+2.4
FSTL1	-1.5	ne	+3.4
FSTL3	-1.5	-2.3 (ns)	+8.8
TAP1	-1.7	nc	+6.8
TAP2	-1.8	nc	+4.6
HPGD	-1.5	ne	+10.5
PLAC8	-1.6	-3.6 (ns)	+11.0
TGFB2	-1.9	ne	+11.8
NOG	-1.9	ne	+4.6
MMP11	-1.7	-8.8	+3.8
SMAD3	-1.6	nc	+4.6
GPRC5A	-2.1	-4.7	+3.8
NUAK1	-1.9	-1.5	+2.4
NUAK2	-1.4	nc	+2.9
AOC1	-1.4	nc	+5.7
NOTCH2	-1.3	nc	+2.4
TEAD1	-1.4	nc	+2.7
ITGA5	-1.3	nc	+7.6
HLA-G	-1.3	-3.7	+10.0
MMP2	-1.2	-7.3	+11.0

Progenitor marker	up in		down in HLAG+
Gene name	CTBs TAZsi	JEG-3 TAZ KO	TBs HLA-G+ vs EGFR+
TP63	+1.7	-3.3	-4.8
CCNB1	+1.5	nc	+2.8
CDC25B	+1.6	nc	nc
CDH5	+2.0	-1.6	+4.4
CDK2	+1.5	nc	-1.5 (ns)
CDK6	+1.7	+1.9	-3.8
MSX2	+2.3	+1.6	-4.0
MTSS1	+3.5	ne	-4.5
OVOL1	+1.6	+1.8	-2.4
SMAD7	+1.5	nc	-1.6 (ns)
SPINT1	+1.8	+1.5	-11.3
TFCP2L1	+3.5	+1.8	-4.8

ns not sig.
nc not changed
ne not expressed
(-) FC **down**
(+) FC **up**

C

STB marker	up in	
Gene name	CTBs TAZsi	JEG-3 TAZ KO
CDKN1A	+1.6	+1.8
CGB3	+2.6	nc
CGB5	+2.5	nc
CGB7	+2.0	nc
CGB8	+2.4	nc
CSH2	+1.7	ne
CYP19A1	+2.5	nc
DUSP9	+2.3	+1.9
ENDOU	+3.6	-2.0
ERVFRD-1	+3.3	-1.4 (ns)
ERVW-1	+2.1	-1.8
GABRE	+1.9	+1.8
GSTA3	+2.5	ne
HSD11B2	+2.9	+2.3 (ns)
SDC1	+1.7	+2.3
TBX3	+2.4	+1.6
TEAD3	+1.5	nc

D

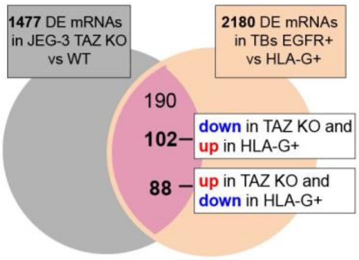

E

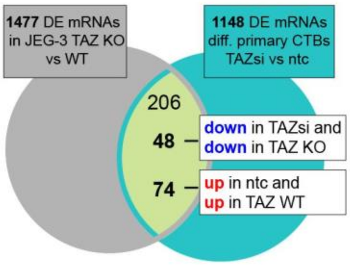

F

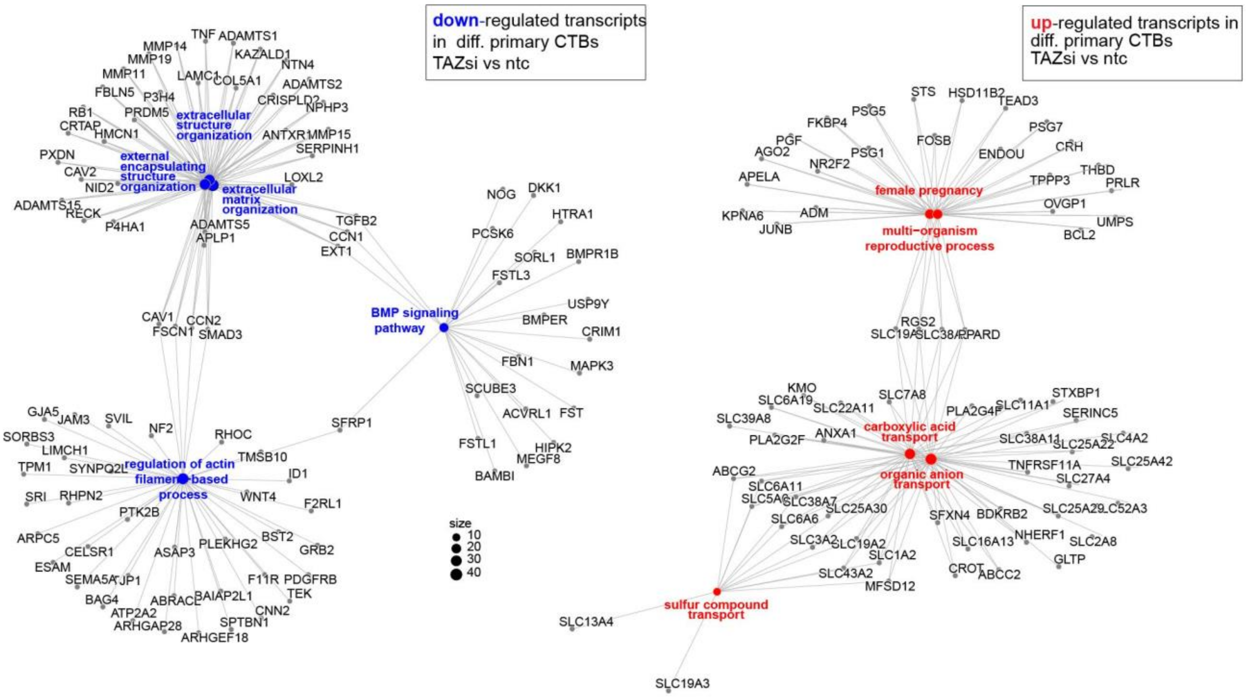

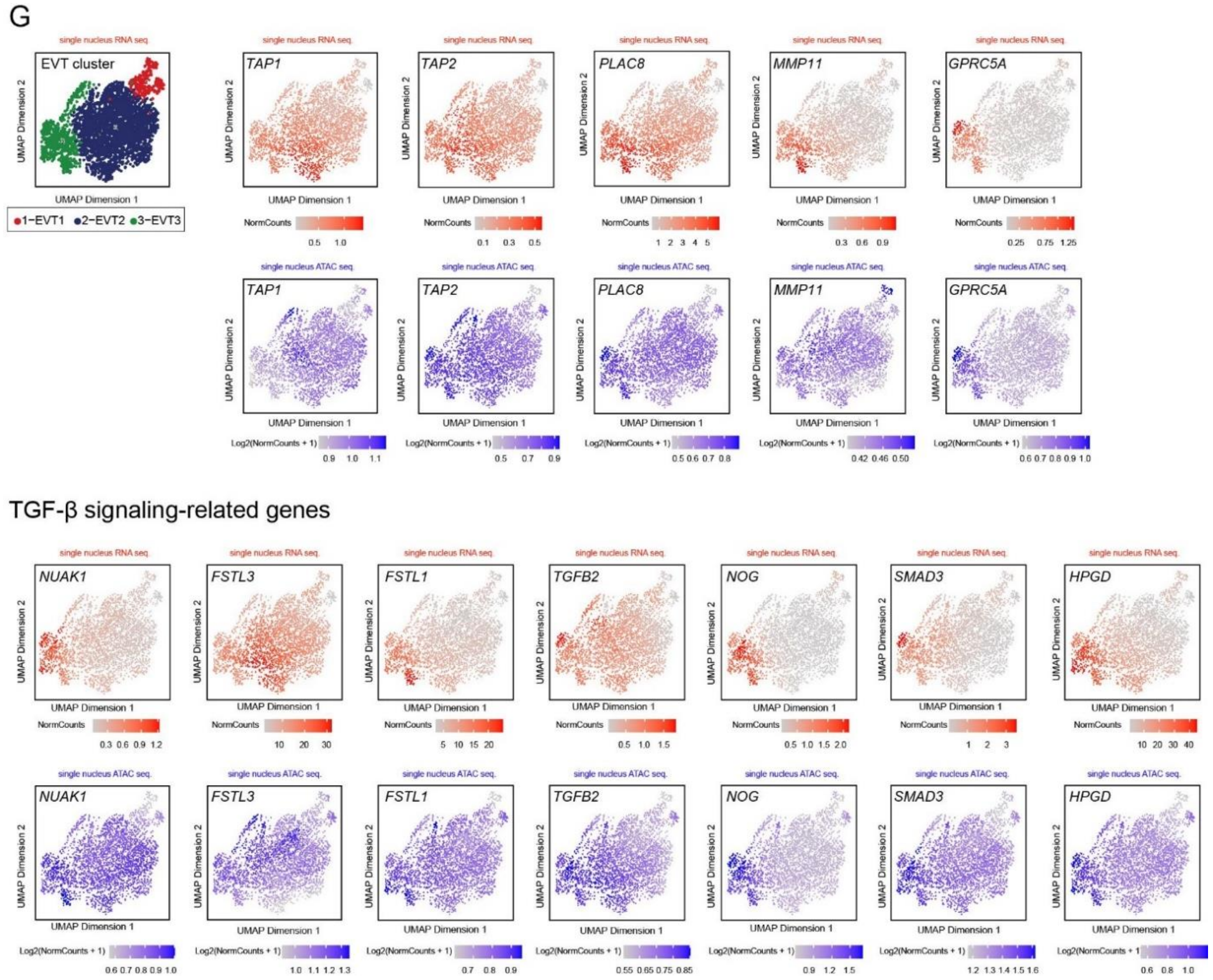

Fig. S3. DEGs, selected transcripts and gene networks in TAZ siRNA-treated primary CTBs and JEG-3 CRISPR-Cas9 genome-edited TAZ knock-out (KO) clones based on bulk RNA-seq data and visualization in EVT clusters of single nuclear RNA-seq and single nuclear ATAC-seq data. (A) Venn diagram showing the overlap of 1148 DEGs obtained from analyses of TAZ siRNA- vs. ntc-treated CTBs (n=3, prepared from 2-3 pooled first trimester placentae 72 hours on fibronectin) with 2180 DEGs established by the comparison of HLA-G$^+$ with EGFR$^+$ primary CTBs as previously published (9). (B) Illustration of the fold changes of DEGs between TAZ siRNA vs. ntc-treated primary CTBs, JEG-3 TAZ KO (n=2) vs. WT (n=2) clones and HLA-G$^+$ vs. EGFR$^+$ primary cells. Selected EVT (left table) and CTB progenitor/stemness markers (right table) are shown. Values of *NUAK1*, *AOC1*, *NOTCH2*, *TEAD1*, *ITGA5*, *HLA-G* and *MMP-2* (left table, first column) show significantly changed transcripts that were below the fold change of < 1.5. (C) Table showing selected STB markers (fold changes) that are upregulated in TAZ siRNA-treated primary CTBs and TAZ KO cells. (D) Venn diagram delineating common genes between DEGs of JEG-3 TAZ KO vs. WT clones and DEGs of HLA-G$^+$ vs. EGFR$^+$ primary trophoblasts. (E) Venn diagram showing the comparison between DEGs of TAZ siRNA vs. ntc with DEGs of JEG-3 TAZ KO vs. WT clones. (F) Gene concept network analysis of TAZsiRNA-incubated primary CTBs. The top 5 statistically significant ontology terms are shown. Enrichment analysis and network creation were performed by using clusterProfiler. (G) UMAP analyses of single-cell (sc) nuclear RNA-seq and sc nuclear ATAC-seq data showing mRNA expression and open chromatin of TAZ target genes in the previously identified EVT clusters of first trimester placentae (15).

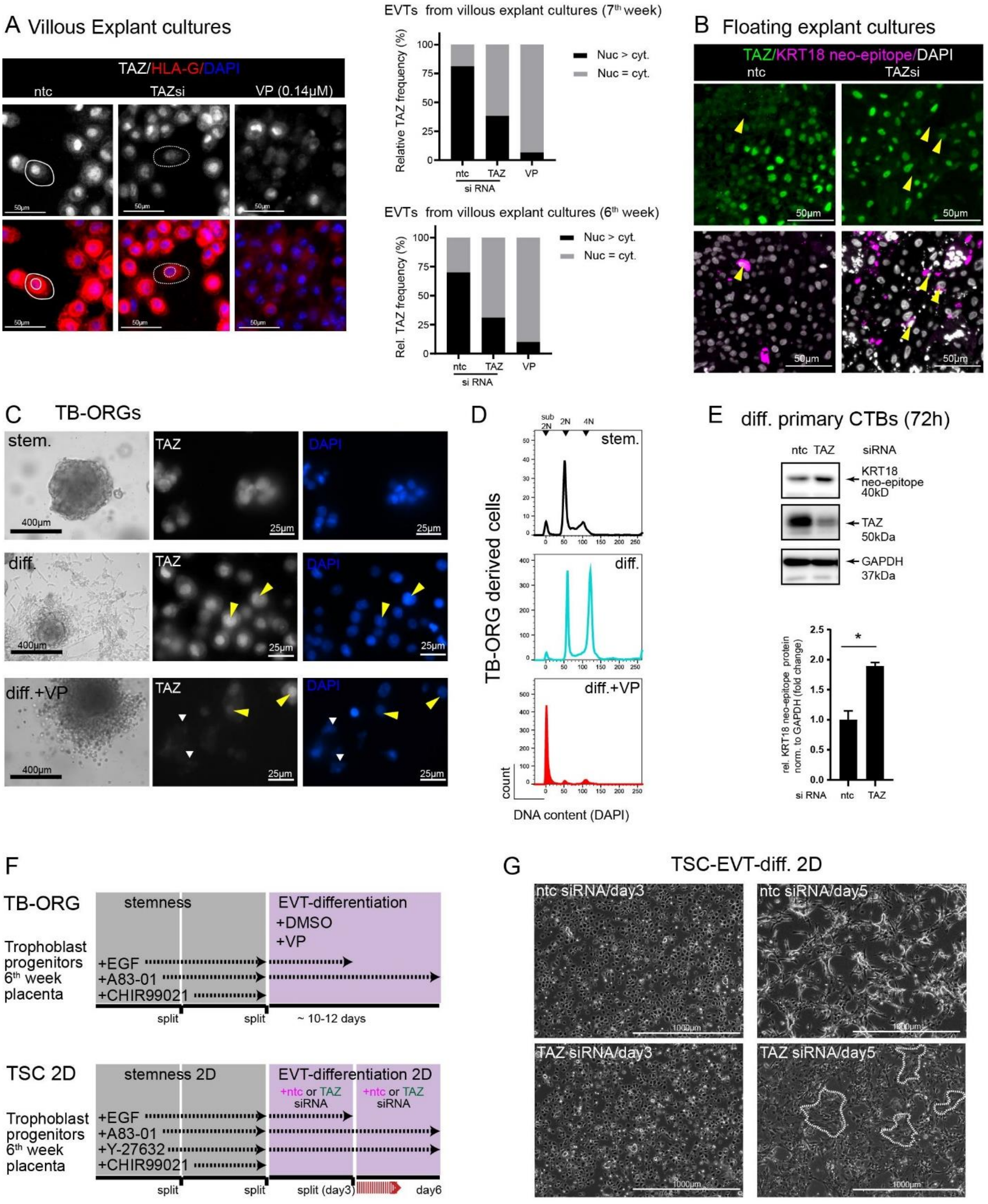

Fig. S4. siRNA-mediated downregulation or chemical inhibition of TAZ impairs EVT differentiation, migration and survival. (A) Representative whole-mount IF pictures of a villous explant cultures (7th week placenta) on collagen I treated with either TAZ siRNAs, non-targeting controls (ntc) or verteporfin (VP) for 72 hours. HLA-G expression in individual EVTs correlates with the abundance of TAZ in their nuclei (examples encircled by dashed lines). Bar graph to the right shows the relative TAZ frequency in the nucleus (nuc.) and cytoplasm (cyt.) of the different treatments derived from two different cultures (6th and 7th week). For each condition between 150 and 200 cells were evaluated. (B) IF of a representative

floating villus explant culture (6th week) showing apoptotic EVTs after incubation with ntc or TAZ siRNAs for 72 hours. KRT18 neo-epitope^{+} EVTs with faint or absent TAZ staining are marked with arrowheads. (C) Light microscopy images of TB-ORGs and IF staining of cells isolated from these cultures (n=3). VP-treated TB-ORGs display EVTs with roundish, apoptotic morphology and contain cells with very faint nuclear TAZ expression (indicated by white arrowheads). Yellow arrowheads mark TAZ^{+} nuclei of unaffected EVTs. DAPI stains nuclei. (D) Flow cytometry of VP-treated TB-ORGs. DNA content (DAPI staining) of self-renewing (black profile) TB-ORGs and cultures differentiated in the absence (turquois profile) and presence of VP (red profile) are shown. (E) Representative immunoblot depicting protein expression in lysates of CTBs (n=3, each pooled from 3-4 6th to 8th week tissues) differentiating on fibronectin for 72 hours in the presence of TAZ or ntc siRNAs. GAPDH was used as loading control. Bar graph shows mean values ± S.D. normalized to GAPDH. *, $p<0.05$. (F) Schematic presentation of the time lines used for siRNA and VP treatment of differentiating TSC and TB-ORGs, respectively. The time window for live cell imaging and cell tracking analyses of TSCs is indicated in red. (G) Representative light microscopy images of ntc- and TAZ siRNA-treated TSCs at day 3 and 5 of differentiation. Dashed lines mark areas with STB morphology.

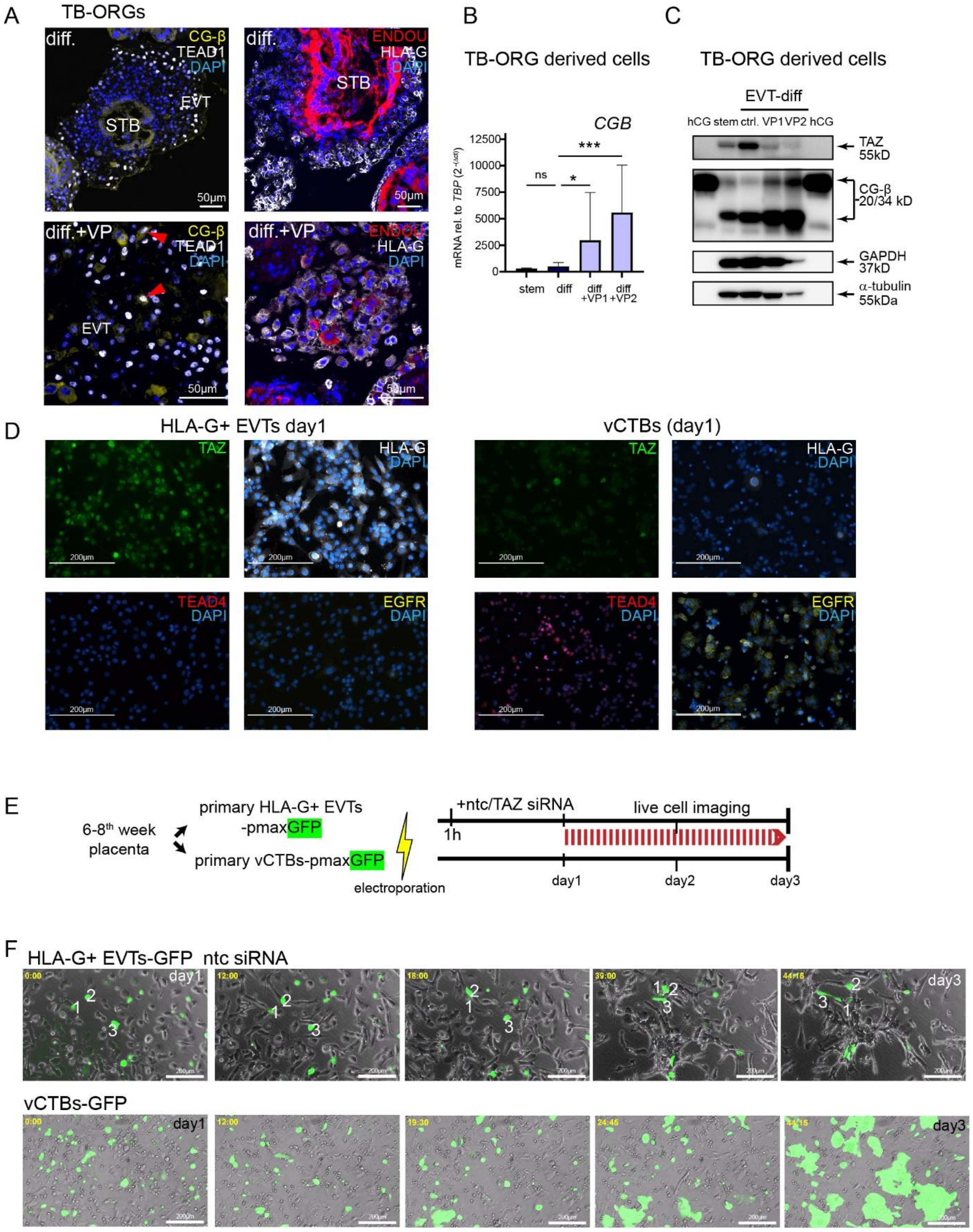

Fig. S5. Characteristics of VP-treated TB-ORGs and GFP-transfected first trimester primary cells. (A) Representative immunofluorescence pictures showing expression of the STB-marker ENDOU in HLA-G^{+} regions of differentiating (diff.) verteporfin (VP)-treated TB-ORGs and CGβ induction in TEAD1^{+} EVTs of these cultures. DAPI stains nuclei. EVT, extravillous trophoblast; STB, syncytiotrophoblast; Mononuclear EVTs expressing CG-β are marked with red arrows. (B) Quantification of *CGB* transcript

levels in TB-ORGs (n=4, prepared from single 6th to 7th week placentae) cultivated under different conditions. Mean values (normalized to TBP) ± SEM, measured in duplicates, are shown. ***, p<0.0002; *, p<0.05. (C) Representative Western blot (n=3) showing TAZ and CGβ levels in lysates of diff. VP-treated TB-ORGs. Urinary human hCG (huCG) was used as positive control. GAPDH and tubulin represent loading controls. (D) Immunofluorescence of HLA-G-purified primary EVTs and vCTBs one day after seeding onto fibronectin. EVTs lacked the CTB-markers EGFR and TEAD4 and expressed HLA-G in more than 99,5 % of cells. (E) Schematic presentation showing the time line for GFP/siRNA transfection and live cell imaging of HLA-G-purified EVTs and vCTBs. (F) Representative light microscopy images of GFP-expressing, non targeting control (ntc) siRNA-transfected primary EVTs (upper panel) and of GFP-expressing vCTBs (lower panel). Despite temporary contacts, cell fusion of GFP-labeled EVTs (1, 2 and 3) could not be observed. In contrast, non-transfected vCTBs form large GFP^{+} STBs at day 3 of cultivation.

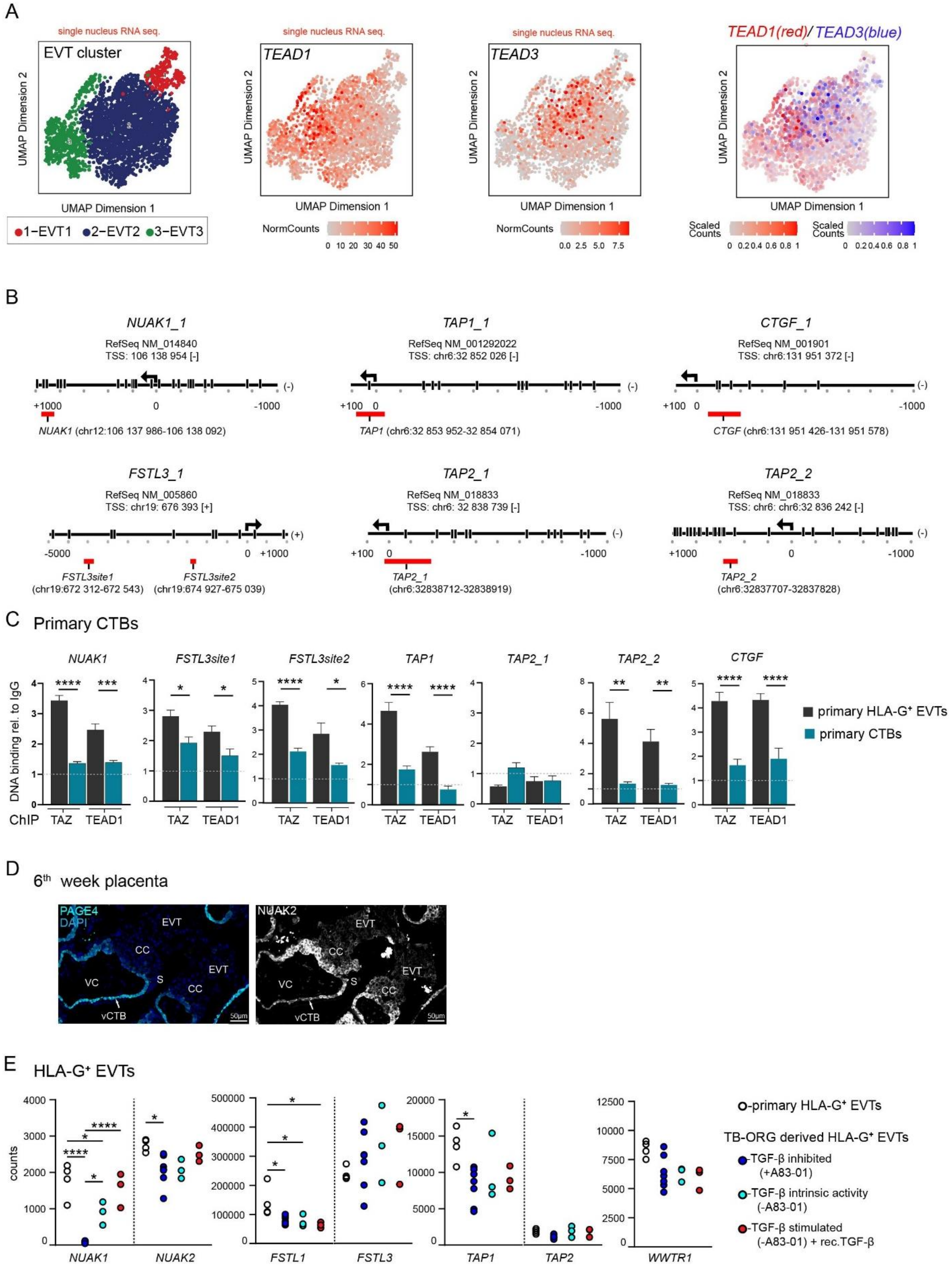

Fig. S6. *TEAD1* and *TEAD3* expression in EVT clusters, identification of TEAD1-TAZ binding sites in selected TAZ-controlled genes and regulation of the latter by TGF-β. (A) UMAP analyses of single-cell (sc) nuclear RNA-seq data showing mRNA expression of *TEAD1* and *TEAD3* in the previously identified

EVT clusters of first trimester placentae (15). (B) Schematic illustration of the promoter regions of selected TAZ target genes based on availability in the Eukaryotic promoter database (4). Chromosomal (chr) orientation of genes and amplicons (in red), harboring TEAD1 binding sites (black vertical lines), are depicted. TSS, transcriptional start site; (C) ChIP-qPCR using two different chromatin pools isolated from HLA-G$^+$ EVTs and HLA-G$^-$ CTBs, respectively. For each pool, chromatin was combined from three different primary cell preparations (total of 23 6th to 10th week placentae). For qPCR different primer sets harboring TEAD1 binding sites were selected. Binding of TEAD1 and TAZ was normalized to ChIP of IgG control. Correct PCR products for FSTL3 were obtained exclusively from regions neighboring TEAD1 recognition sequences. The amplicon in the *TAP2_1* promoter did not shown TEAD1 or TAZ binding. Mean values ± SEM, measured in triplicates, are shown. ****, p<0.0001; ***, p<0.0002; **, p<0.002; *, p<0.05; (D) Representative immunofluorescence images showing NUAK2 localization in first trimester placenta (n=3). Stemness marker PAGE4 marks proliferative CTB populations. CC, cell column; EVT, extravillous trophoblast; S, syncytium; VC, villous core; vCTB, villous cytotrophoblast; (E) Transcript levels of *WWTR1*, encoding TAZ, and expression of selected TAZ target genes in the previously established bulk RNA-seq data of HLA-G$^+$ EVTs purified from TB-ORGs (1). EVTs were generated upon removal of the WNT activator CHIR99021 and further differentiated in the absence or presence of TGF-β signalling. Single values of TGF-β-activated (n=3) and TGF-β-inhibited (n=6) EVT samples are depicted. For comparison measures of isolated HLA-G$^+$ pEVTs (n=4) are shown. Only significant changes are depicted. ****p<0.0001; *p<0.05;

SI Appendix Movies

Movie S1. Migration of first trimester placenta-derived TSCs on fibronectin that were differentiated into EVTs and treated with non-targeting control siRNAs as outlined in SI Appendix, Fig. S4F. Live cell imaging was performed for 49 hours after day 3 of differentiation using Lionheart FX Automated Microscope.

Movie S2. Migration of first trimester placenta-derived TSCs on fibronectin that were differentiated into EVTs and treated with TAZ siRNAs as indicated in SI Appendix, Fig. S4F. Live cell imaging was performed for 49 hours after day 3 of differentiation using Lionheart FX Automated Microscope.

Movie S3. Live cell imaging of HLA-G^+-purified first trimester primary EVTs on fibronectin. Cells were transfected with GFP plasmids and non-targeting control siRNAs as shown in SI Appendix, Fig. S5E. Imaging was performed for 44 hours using Lionheart FX Automated Microscope, one day after seeding and genetic manipulation.

Movie S4. Live cell imaging of HLA-G^+-purified first trimester primary EVTs on fibronectin. Cells were transfected with GFP plasmids and TAZ siRNAs as depicted in SI Appendix, Fig. S5E. Imaging was performed for 44 hours using Lionheart FX Automated Microscope, one day after seeding and genetic manipulation.

Movie S5. Live cell imaging of purified first trimester villous CTBs on fibronectin. Cells were transfected with GFP plasmids as indicated in SI Appendix, Fig. S5E. Imaging was performed for 44 hours using Lionheart FX Automated Microscope, one day after seeding and GFP transfection.

SI Appendix Datasets

Dataset S1. The dataset shows the list of genes differentially expressed between TAZ siRNA- and non-targeting control (ntc)-treated primary CTBs after 72 hours of EVT differentiation on fibronectin. Values of six gene silenced cell pools, each derived from 2-3 pooled 8th to 10th week placentae, are depicted.

SI Appendix References

1. S. Haider *et al.*, Transforming growth factor-beta signaling governs the differentiation program of extravillous trophoblasts in the developing human placenta. *Proc Natl Acad Sci U S A* **119**, e2120667119 (2022).
2. F. Piccinini, A. Kiss, P. Horvath, CellTracker (not only) for dummies. *Bioinformatics* **32**, 955-957 (2016).
3. S. Kolmykov *et al.*, GTRD: an integrated view of transcription regulation. *Nucleic Acids Res* **49**, D104-D111 (2021).
4. R. Cavin Perier, T. Junier, P. Bucher, The Eukaryotic Promoter Database EPD. *Nucleic Acids Res* **26**, 353-357 (1998).
5. I. Yevshin, R. Sharipov, T. Valeev, A. Kel, F. Kolpakov, GTRD: a database of transcription factor binding sites identified by ChIP-seq experiments. *Nucleic Acids Res* **45**, D61-D67 (2017).
6. S. W. Wingett, S. Andrews, FastQ Screen: A tool for multi-genome mapping and quality control. *F1000Res* **7**, 1338 (2018).
7. A. Dobin *et al.*, STAR: ultrafast universal RNA-seq aligner. *Bioinformatics* **29**, 15-21 (2013).
8. M. I. Love, W. Huber, S. Anders, Moderated estimation of fold change and dispersion for RNA-seq data with DESeq2. *Genome Biol* **15**, 550 (2014).
9. S. Vondra *et al.*, Metabolism of cholesterol and progesterone is differentially regulated in primary trophoblastic subtypes and might be disturbed in recurrent miscarriages. *J Lipid Res* **60**, 1922-1934 (2019).
10. T. Hulsen, J. de Vlieg, W. Alkema, BioVenn - a web application for the comparison and visualization of biological lists using area-proportional Venn diagrams. *BMC Genomics* **9**, 488 (2008).
11. R. S. Blighe K, Lewis M (2023) EnhancedVolcano: Publication-ready volcano plots with enhanced colouring and labeling. R package version 1.18.0.
12. G. Yu, L. G. Wang, G. R. Yan, Q. Y. He, DOSE: an R/Bioconductor package for disease ontology semantic and enrichment analysis. *Bioinformatics* **31**, 608-609 (2015).
13. S. Draghici *et al.*, A systems biology approach for pathway level analysis. *Genome Res* **17**, 1537-1545 (2007).
14. M. Kanehisa, S. Goto, KEGG: kyoto encyclopedia of genes and genomes. *Nucleic Acids Res* **28**, 27-30 (2000).
15. J. R. Ounadjela *et al.*, Spatial multiomic landscape of the human placenta at molecular resolution. *Nat Med* 10.1038/s41591-024-03073-9 (2024).
16. B. Li *et al.*, Cumulus provides cloud-based data analysis for large-scale single-cell and single-nucleus RNA-seq. *Nat Methods* **17**, 793-798 (2020).
17. J. M. Granja *et al.*, ArchR is a scalable software package for integrative single-cell chromatin accessibility analysis. *Nat Genet* **53**, 403-411 (2021).
